# Supplementary material for: Proprioceptive accuracy in Immersive Virtual Reality: A developmental perspective
Source: PLoS One. 2020 Jan 30;15(1):e0222253. doi: 10.1371/journal.pone.0222253 (PMC6992210; doi:10.1371/journal.pone.0222253)
Supplement: S1 Appendix — (PDF) [file pone.0222253.s001.pdf]

Proprioceptive Accuracy in Immersive Virtual Reality: A Developmental Perspective

Irene Valori, Phoebe E. McKenna-Plumley, Rena Bayramova,
Claudio Zandonella Callegher, Gianmarco Altoè & Teresa Farroni

Supplemental Materials

Edited by

Claudio Zandonella Callegher* & Gianmarco Altoè

*e-mail address
claudiozandonella@gmail.com

January 15, 2020

Contents

1 Introduction **1**
1.1 Statistical Approach . . . 1
1.2 Statistical Analysis . . . 2

2 Data Presentation **3**

3 Descriptive Statistics **5**
3.1 Subjects . . . 5
3.2 Number of Observations . . . 5
3.3 Amplitude . . . 6
3.3.1 Variability of Amplitude across conditions and Age groups . . . 7
3.4 Self-turn Error . . . 8

4 Model Comparison **11**
4.1 Models Specification . . . 11
4.1.1 Generalized Linear Model . . . 12
4.2 Models Priors . . . 12
4.3 Models Estimation . . . 13
4.3.1 `m.0` . . . 14
4.3.2 `m.1` . . . 15
4.3.3 `m.2` . . . 17
4.3.4 `m.3` . . . 19
4.3.5 `m.4` . . . 21
4.3.6 `m.5` . . . 23
4.3.7 `m.6` . . . 25
4.4 Model Comparison . . . 27

5 Description of Model `m.2` **29**
5.1 Model Parameters . . . 29
5.1.1 Amplitude . . . 30
5.1.2 Age . . . 30
5.1.3 Interaction between Perception and Environment . . . 32
5.2 Effect Sizes . . . 33
5.3 Model Fit . . . 34

6 Maximum Likelihood **36**

7 Session Information **37**

Bibliography **39**

List of Figures

1 Amplitude distribution 6
2 Variability of Amplitude according to conditions and Age groups 7
3 Frequency of Self-turn errors 9
4 Distributions of Self-turn errors 10
5 Parameters prior distributions 12
6 Parameters trace plots `m.0` 14
7 Parameters trace plots `m.1` 15
8 Parameters trace plots `m.2` 17
9 Parameters trace plots `m.3` 19
10 Parameters trace plots `m.4` 21
11 Parameters trace plots `m.5` 23
12 Parameters trace plots `m.6` 25
13 Predicted mean of Self-turn error according to Amplitude.st 30
14 Distributions of the predicted means of Self-turn error according to Age 31
15 Distributions of the predicted means of Self-turn error according to the experimental conditions 32
16 Posterior predictive check 35

List of Tables

1 Number of subjects for each Age group. 5
2 Observations for each subject. 5
3 Number of observations according to Age groups and conditions. 6
4 Descriptive statistics 8
5 Model formulas 11
6 R-hat values model `m.0` 14
7 R-hat values model `m.1` 16
8 R-hat values model `m.2` 18
9 R-hat values model `m.3` 20
10 R-hat values model `m.4` 22
11 R-hat values model `m.5` 24
12 R-hat values model `m.6` 26
13 WAIC model comparison 27
14 Estimated parameters of model `m.2` 29
15 Predicted means and differences of Self-turn error according to age. 31
16 Predicted means and differences of Self-turn error according experimental conditions. 33
17 Effect size as the ratio of the scores 34
18 Bayesian R^2 35
19 Comparison between Bayesian `m.2` and Maximum-Likelihood `glm.2` models 36

1 Introduction

In this report the statistical analyses of the article *"Proprioceptive Accuracy in Immersive Virtual Reality: A Developmental Perspective"* are presented. The aim of the study is to investigate the extent to which the reliability of visual information aids proprioceptive-based self-motion accuracy across the developmental lifespan. Moreover, it explores whether Immersive Virtual Reality (IVR) environments, compared to equivalent real environments, affect proprioceptive accuracy.

Here, we will focus only on the statistical analyisis. For theoretical aspects, study aims, experiment design, and results interpretation the reader can refer directly to the article.

1.1 Statistical Approach

In order to evaluate the research hypotheses about how Age, Perception, and Environment interact to affect self-motion accuracy, a model comparison approach was used.

Model comparison allows for the selection of the most plausible models given the data and a set of candidate models (McElreath, 2016). Models are compared in terms of statistical evidence (i.e. support by the data) using information criteria (Wagenmakers & Farrell, 2004), which enables the evaluation of models considering the trade-off between parsimony and goodness-of-fit (Vandekerckhove, Matzke, & Wagenmakers, 2015): as the complexity of the model increases (i.e. more parameters) the fit to the data increases as well but generalizability (i.e. ability to predict new data) decreases. The researchers' aim is to find the right balance between fit and generalizability in order to describe, with a statistical model, the important feature of the studied phenomenon, but not the random noise of the observed data. Information criteria provide an estimate of the average deviance (i.e. error) of the model's ability to predict new data, thus lower values are interpreted as indications of a better model (McElreath, 2016).

Given the complex structure of the data, Bayesian Generalized Mixed-Effects Models are used. Specifically, data are characterized by: (1) a continuous non-normally distributed dependent variable (i.e. rotation error); (2) a between-subject factor (i.e. age); (3) within-subject factors (i.e. Perception and Environment); (4) a quantitative independent variable (i.e. rotation Amplitude).

- **Mixed-Effects Models.** Mixed-Effects Models allow us to take into account the repeated measures design of the experiment (i.e. observations nested within subjects). Thus, participants are treated as random effects, with random intercepts that account for the interpersonal variability, whereas the other variables are considered as fixed effects.

- **Generalized Linear Models.** Generalized linear models were used considering the Gamma distribution, with logarithmic link function, as the probability distribution of the dependent variable. Generalized linear models allow to model non-normally distributed data using appropriate probability distributions that reflect the characteristics of the data (Fox, 2016). Selecting an appropriate probability distribution provides better fit to the data and more reliable results (Lo & Andrews, 2015). Gamma distribution is advised in the case of positively skewed, non-negative data, when the variances are expected to be proportional to the square of the means (Ng & Cribbie, 2017). These conditions are respected by our dependent variable: we only have positive values, with a positive skewed distribution, and we expect a greater variability of the possible results as the model predicted mean increases (i.e. a greater dispersion of subjects' scores when greater mean values are predicted by the model).

- **Bayesian approach.** The Bayesian approach is a valid alternative to the traditional frequentist approach (Gelman et al., 2013; Kruschke & Liddell, 2018) and in the case of mixed-effects models it allows for the accurate estimation of complex models that otherwise would fail to converge (i.e. unreliable results) in a traditional frequentist approach (Bolker et al., 2009; Fong, Rue, & Wakefield, 2010). Without going into philosophical reasons, which are beyond the scope of the present paper (if interested consider Romeijn & van de Schoot, 2008), Bayesian inference has some unique elements that make the meaning and interpretation of the results different from the classical frequentist approach (Etz & Vandekerckhove,

2018). In particular, in the Bayesian approach parameters are estimated using probability distributions (i.e. a range of possible values) and not a single point estimate (i.e. a single value). Bayesian inference has three main ingredients (van de Schoot et al., 2014): (1) *Priors*, the probability distributions of possible parameter values considering the information available before conducting the experiment; (2) *Likelihood*, the information given by the observed data about the probability distributions of possible parameter values; (3) *Posteriors*, the resulting probability distributions of possible parameter values, obtained by combining Priors and Likelihood through Bayes' Theorem. As a result, the Bayesian approach assesses the variability (i.e. uncertainty) of parameter estimates and provides associated inferences via 95% Bayesian Credible Intervals (BCIs): the range of most credible parameter values given the prior distribution and the observed data. Thus, the Bayesian approach allows for the description of the phenomenon of interest through probabilistic statements, rather than a series of simplified reject/not-reject dichotomous decisions typically used in the null hypothesis significance testing approach (McElreath, 2016).

Analysis were conducted with the R software version 3.6.1 (R Core Team, 2018). Models were estimated using the R package `brms` (Bürkner, 2017) which is based on STAN programming language (Stan Development Team, 2017a, 2017b) and employs the No-U-Turn Sampler (NUTS; Hoffman & Gelman, 2014), an extension of Hamiltonian Monte Carlo (Neal, 2012). The basic idea of estimating parameters through a sampling procedure is to iteratively poll possible parameter values from pre-specified prior distributions. Parameter values that support the data are rewarded, whereas those values that do not support the data are penalized. After a large number of iterations convergence upon those model parameters that optimally represent the data is obtained. Iterations of the estimation procedure are split among independent chains (i.e. independent sampling processes) to ensure that the model reliably converges on the same parameter values. Since each chain has a random starting point, it requires a certain number of iterations before reaching an optimal convergence. Thus, for each chain the initial samples, known as *"warm-up"*, are discarded and only the subsequent sampling part is used for inference purposes.

1.2 Statistical Analysis

The analysis is divided into different sections:

- **Section 2.** We introduce the dataset with a brief description of each variable.
- **Section 3.** We present the descriptive statistics of our dataset.
- **Section 4.** We formalize the models and present the prior and the diagnostic criteria for convergence of parameters estimation. Finally, we give the results of the model comparison.
- **Section 5.** The most plausible model is interpreted using the estimated posterior parameter distributions. Main effects and interaction effects are considered using planned comparison and graphical representations of the predicted values by model. Finally, we evaluate the model fit considering R^2 value and model Posterior Predictive check.
- **Section 6.** We present a separate set of analyses where all models are estimated with the traditional maximum likelihood approach using the R package `lme4` (Bates, Mächler, Bolker, & Walker, 2014). In several cases, convergence is not reached (i.e. unreliable results). Overall, estimated model parameters are very similar to those produced by the Bayesian approach.

2 Data Presentation

In the present dataset we have 13 variables:

1. **Id**. Each subject has an unique Id ranging from 1 to 49.
2. **Years**. The age in years of each subject.
3. **Age**. We have three different age groups: *Adults* 18-45 years; *Older Children* subjects between 9 and 15 years old; *Young Children* subjects 4 and 8 years old and younger.
4. **Environment**. The two environmental conditions *Reality* (*R*) and *Immersive Virtual Reality* (*IVR*).
5. **Perception**. The three sensory conditions *Proprioception* (*P*), *Vision* (*V*), and *Vision + Proprioception* (*VP*).
6. **Condition**. A summary index between 1 and 6 to specify the experimental condition.
   - *1*) Reality and Proprioception
   - *2*) Reality and Vision
   - *3*) Reality and Vision + Proprioception
   - *4*) IVR and Proprioception
   - *5*) IVR and Vision
   - *6*) IVR and Vision + Proprioception
7. **Direction**. The direction of the passive rotation imposed by the experimenter on the subject, *Right* (*R*) or *Left* (*L*).
8. **Amplitude**. The amplitude of the passive rotation imposed by the experimenter on the subject, *Big* approximately 180 degrees or *Small* approximately 90 degrees.
9. **Start.Position**. Position from which the experimenter started the passive rotation.
10. **End.Position**. Position where the experimenter stopped the passive rotation.
11. **Return.Position**. Position in which the subject stopped the active self-turn.
12. **Angle**. The actual angle of the passive rotation imposed by the experimenter on the subject.
13. **Error**. The absolute error committed by the subject in the Self-turn task, computed as the absolute rotation difference between *Start.Position* and *Return.Position*.

Below we report the structure of the dataset.

```
load(paste0(data_directory,"data.rda"))

str(d)

## 'data.frame': 578 obs. of  13 variables:
##  $ Id             : Factor w/ 49 levels "1","2","3","4",..: 1 1 1 1 1 1 1 1 1 1 ...
##  $ Years          : int  13 13 13 13 13 13 13 13 13 13 ...
##  $ Age            : Factor w/ 3 levels "Adults","Older_Children",..: 2 2 2 2 2 2 2 2 2 2 ...
##  $ Environment    : Factor w/ 2 levels "R","IVR": 1 1 2 2 1 1 2 2 2 2 ...
##  $ Perception     : Factor w/ 3 levels "P","V","VP": 3 3 3 3 1 1 2 2 1 1 ...
##  $ Condition      : Factor w/ 6 levels "1","2","3","4",..: 3 3 6 6 1 1 5 5 4 4 ...
##  $ Direction      : Factor w/ 2 levels "R","L": 1 2 2 1 1 2 2 1 1 2 ...
```

```
##  $ Amplitude      : Factor w/ 2 levels "Big","Small": 1 2 1 2 1 2 1 2 1 2 ...
##  $ Start.Position : num  225 225 225 228 233 217 220 209 215 230 ...
##  $ End.Position   : num  49 133 46 310 20 140 49 315 18 145 ...
##  $ Return.Position: num  225 229 228 235 218 220 209 215 230 231 ...
##  $ Angle          : num  184 92 179 82 147 77 171 106 163 85 ...
##  $ Error          : num  0 4 3 7 15 3 11 6 15 1 ...
```

3 Descriptive Statistics

3.1 Subjects

Considering the Age of the subjects we have 23 *Adults* subjects, 13 *Older Children* subjects, and 13 *Young Children* subjects (1).

Table 1: Number of subjects for each Age group.

**age**	**n.subjects**	**percentages**
Adults	23	0.47
Older Children	13	0.27
Young Children	13	0.27
Total	49	1.00

3.2 Number of Observations

Out of the 49 participants, 43 subjects completed the task in all 12 conditions, 4 subjects completed 11 conditions, 1 subject completed 10 conditions, and 1 subject completed 8 conditions (2). Thus, the final data consist of 578 observations nested in 49 subjects.

Table 2: Observations for each subject.

**n.subjects**	**n.observations**
1	8
1	10
4	11
43	12

The number of observations according to age groups and experimantal conditions is reported in 3.

Table 3: Number of observations according to Age groups and conditions.

	Proprioception	Vision	Vision + Proprioception	Total
**Reality**				
Adults	46	45	46	137
Older Children	26	26	25	77
Young Children	25	25	25	75
Total	97	96	96	289
**IVR**				
Adults	44	48	46	138
Older Children	26	26	26	78
Young Children	24	25	24	73
Total	94	99	96	289
**Total**				
Adults	90	93	92	275
Older Children	52	52	51	155
Young Children	49	50	49	148
Total	191	195	192	578

Note: IVR = Immersive Virtual Reality. $n_{subjects} = 49; n_{observations} = 578$

3.3 Amplitude

For each condition, the task aimed to include two rotations of different Amplitude, one 180 degree (*Big*) rotation and one 90 degree (*Small*) rotation. However, as we can see in 1, there is a great variability in the actual rotation (reported in the variable **Angle**). Thus, we considered **Amplitude** as a continuous variable and not as a dichotomous categorical variable.

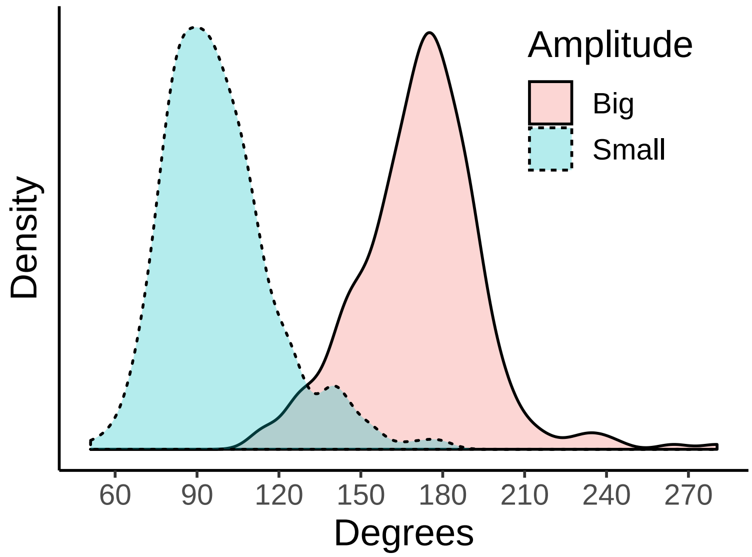

Figure 1: Amplitude distribution of the actual Self-turn rotation task (i.e. task difficulty; $n_{subjects} = 49$; $n_{observations} = 578$).

Moreover, to obtain interpretable results in the analyses, the **Amplitude** variable was standardized (i.e., Z-scores were obtained).

```
d$Amplitude.st<-(d$Angle-mean(d$Angle))/sd(d$Angle)
```

3.3.1 Variability of Amplitude across conditions and Age groups

In 2 the distribution of the actual Self-turn rotation task is presented in the different experimental conditions according to Age groups. It is possible to observe that distributions are slightly different, but all of them cover appropriately the same range of values. Considering the Amplitude as a continuous variable and not as a dichotomous categorical variable guarantees that results are not influenced by the variability in the actual rotation between experimental conditions and Age groups.

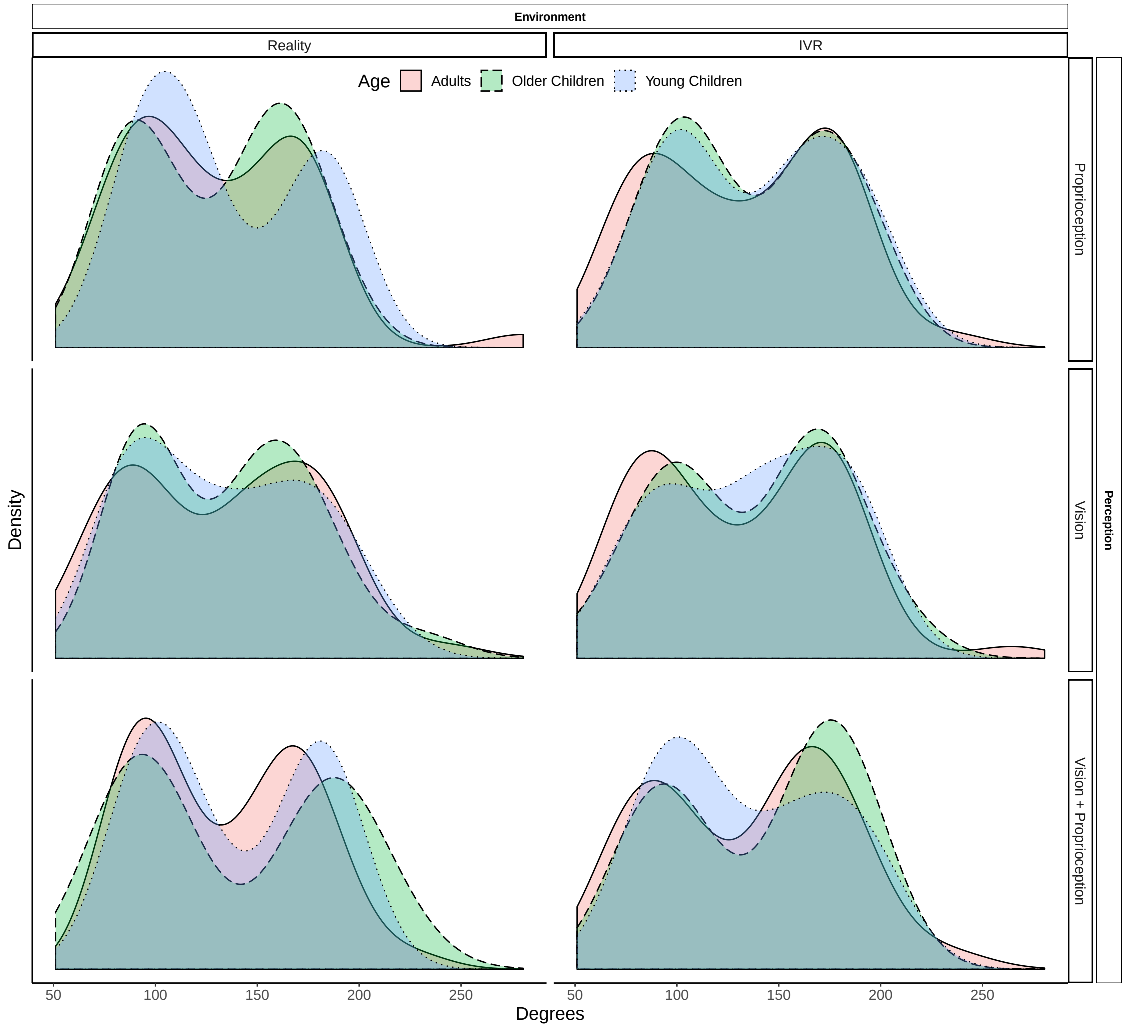

Figure 2: Amplitude distribution of the actual Self-turn rotation in the different experimental conditions according to Age group ($n_{subjects} = 49$; $n_{observations} = 578$).

Table 4: Descriptive statistics. Means and standard deviations of self-turn error according to Age and the experimental conditions

	Proprioception		Vision		Vision + Proprioception		Total	
	**Mean**	**SD**	**Mean**	**SD**	**Mean**	**SD**	**Mean**	**SD**
**Reality**								
Adults	16.2	8.6	9.8	12.6	6.1	4.1	10.7	6.0
Older Children	19.6	10.5	14.0	18.2	6.7	3.6	13.5	7.3
Young Children	30.6	22.4	8.2	5.2	20.7	20.5	19.8	9.0
Total	20.9	15.0	10.5	12.9	10.1	12.5	13.9	8.0
**IVR**								
Adults	17.6	10.6	13.5	7.6	13.7	9.1	14.9	6.3
Older Children	23.6	19.1	17.5	10.1	16.9	18.6	19.3	9.5
Young Children	37.8	16.2	28.5	16.5	25.1	16.5	30.3	9.9
Total	24.7	16.8	18.5	12.6	17.4	14.6	20.2	10.3
**Total**								
Adults	17.1	6.4	11.8	8.0	9.9	4.8	12.8	4.4
Older Children	21.6	13.7	15.7	11.8	11.7	9.4	16.4	7.5
Young Children	34.2	18.0	18.2	7.9	23.4	15.8	25.3	7.7
Total	22.8	14.1	14.5	9.3	13.9	11.3	17.1	8.0

Note: IVR = Immersive Virtual Reality. $n_{subjects} = 49; n_{observations} = 578$

3.4 Self-turn Error

Given that observations are not independent but nested within subjects, to compute descriptive statistics in each condition first the mean Self-turn error was obtained for each subject and then mean and standard deviation were computed.

In the present sample, the mean Self-turn error was 17.07 degrees ($SD = 8.05$).

```
mean(tapply(d$Error,INDEX=d$Id, FUN= mean))

## [1] 17.0724

sd(tapply(d$Error,INDEX=d$Id, FUN= mean))

## [1] 8.046226
```

Mean and standard deviation according to Age groups and the different experimental conditions are reported in 4.

The frequency of the observed values is reported in 3. Since Self-turn error is computed as the absolute difference between *Start.Position* and *Return.Position*, only positive values are possible and from visual inspection the dependent variable has an evident positive skewed distribution (skewness value = 2.49).

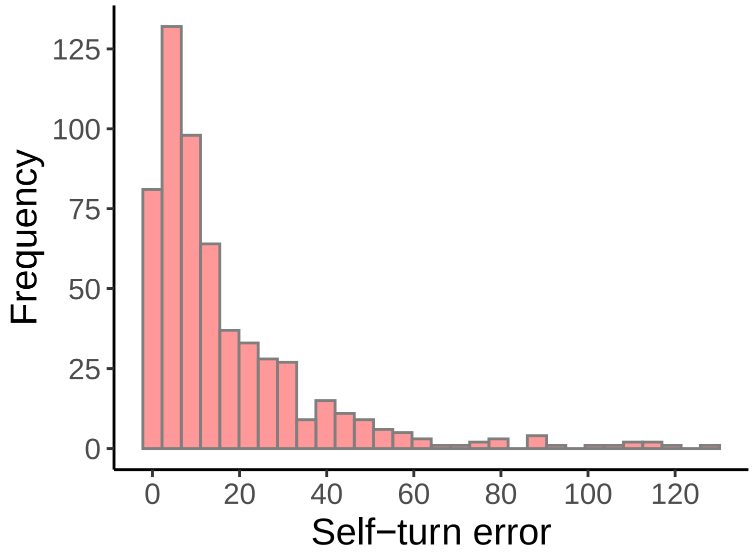

Figure 3: Frequency of the observed Self-turn errors ($n_{subjects} = 49$; $n_{observations} = 578$).

Finally we represent in 4 the distribution of the observed Self-turn error according to age groups and the different experimental conditions.

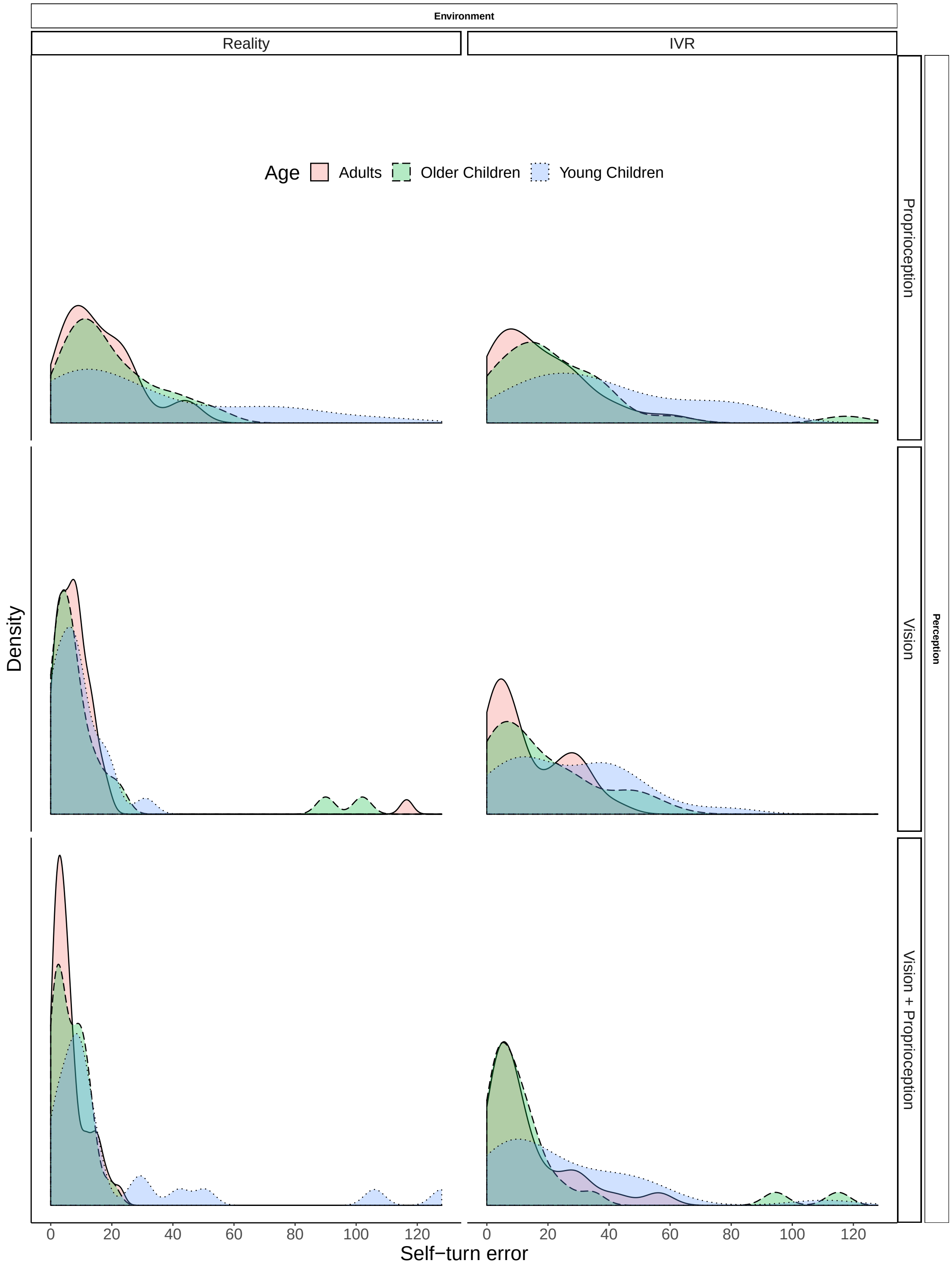

Figure 4: Distributions of the observed Self-turn errors in the different conditions according to Age ($n_{subjects} = 49$; $n_{observations} = 578$).

4 Model Comparison

In this section we first specify all the Bayesian Generalized Mixed-Effects Models used in the analysis. We formalize as statistical models the different research hypotheses, which include 2-way and 3-way interactions between independent variables. Subsequently we present the prior and the diagnostic criteria for convergence of parameters estimation. Finally, we give the results of the model comparison.

4.1 Models Specification

Seven different Bayesian Generalized Mixed-Effects Models were performed to analyse the data. In each model the dependent variable was the error in the self-turn task. The first model (`m.0`) was a baseline model considering the random effect of **Id** (i.e. interpersonal varibility) and the fixed effects of **Direction** (i.e. right or left rotation) and of **Amplitude.st** (i.e. amplitude of the rotation in degrees). This baseline model, that includes the effects of possible confounding variables, was used as a reference point to then evaluate the models that considered the effects of **Age**, **Perception** and **Environment**.

Below, the formula for each model is reported :

$$m.0 : Error \sim Amplitude.st + Direction + (1|Id)$$
$$m.1 : Error \sim Age + Perception + Environment + Amplitude.st + Direction + (1|Id)$$
$$m.2 : Error \sim Age + Perception * Environment + Amplitude.st + Direction + (1|Id)$$
$$m.3 : Error \sim Age * Environment + perception + Amplitude.st + Direction + (1|Id)$$
$$m.4 : Error \sim Age * Perception + Environment + Amplitude.st + Direction + (1|Id)$$
$$m.5 : Error \sim (Age + Perception + Environment)^2 + Amplitude.st + Direction + (1|Id)$$
$$m.6 : Error \sim (Age + Perception + Environment)^3 + Amplitude.st + Direction + (1|Id)$$

In `m.1` the additive effects of Age, Perception and Environment were added to the baseline model. Single 2-way interactions were evaluated in models `m.2`, `m.3`, and `m.4`. In the model `m.5` all the possible 2-way interactions between Age, Perception and Environment were considered together. Finally, in `m.6` also the 3-way interaction was added. Models are summarized in 5.

Table 5: Model formulas

	**Model**	**Dependent Variable**		**Predictors**: **Fixed Effects**	**Predictors**: **Random Effect**
Baseline	`m.0`	error	←	Amplitude.st + Direction	Id
Additive	`m.1`	error	←	m.0 + Age + Perception + Environment	Id
2-way Interactions	`m.2`	error	←	m.1 + Perception × Environment	Id
	`m.3`	error	←	m.1 + Age × Environment	Id
	`m.4`	error	←	m.1 + Age × Perception	Id
All 2-way Interactions	`m.5`	error	←	m.1 + Perception × Environment+ Age × Environment + Age × Perception	Id
3-way Interactions	`m.6`	error	←	m.5 + Age × Perception × Environment	Id

4.1.1 Generalized Linear Model

We specified the Gamma distribution with logarithmic link function as the family distribution for our Bayesian Generalized Mixed-Effects Models. Given that there are some 0 values in the Self-turn error, in order to use the logarithmic link function we need first to add 1 to all the errors. This is done to avoid indefinite values (i.e. $\log(0) = -\infty$).

```
d$Error1<-d$Error+1
```

4.2 Models Priors

All our models used default prior specification of the R package `brms` (Bürkner, 2017) that are considered to be only very weakly informative in order to influence results as little as possible, while providing at least some regularization to considerably improve convergence and sampling efficiency. This allows the posterior distributions to be mostly influenced by the observed data rather than by prior information.

In particular, for the population-level effects (i.e. fixed effects) `brms` uses an improper flat prior over the reals. Group-level effects (i.e. random effects) are restricted to be non-negative and, by default, have a half student-t prior with 3 degrees of freedom and a scale parameter of 10 `student_t(3, 0, 10)`. Intercept prior is set to be a student-t with 3 degrees of freedom, location parameter of 2 and scale parameter of 10 `student_t(3, 2, 10)`. Finally the prior for the shape parameter for the gamma distribution is set to `gamma(0.01, 0.01)`.

In 5 the different prior distributions are presented.

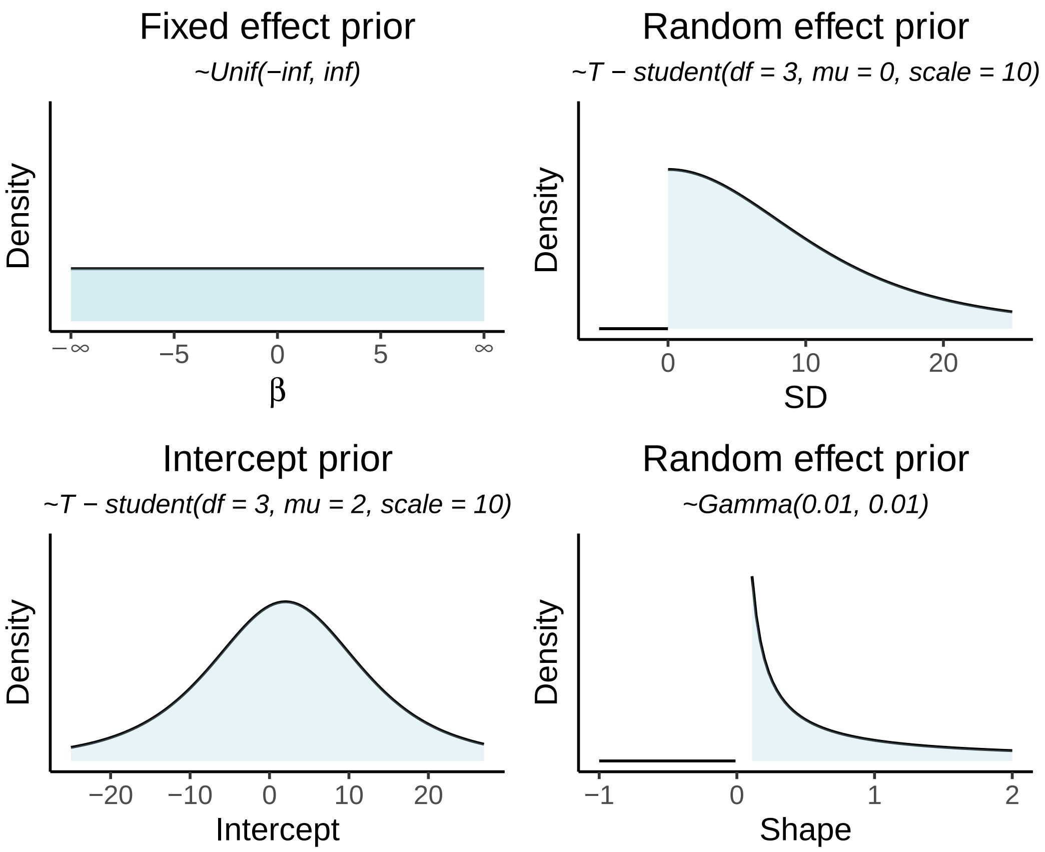

Figure 5: Parameter prior distributions

Remember that by default, the population-level intercept is estimated separately and not as part of population-level parameter vector `b`. Moreover, to increase sampling efficiency, the population-level design matrix `X` is centered around its column means `X_means`. Thus, intercept prior is defined on the centered design matrix and not on the real intercept.

Below we report the default prior settings of brms for `m.0`. For the other models the settings are the same just with more β parameters.

```
get_prior(Error1~  Amplitude.st + Direction + (1|Id),family=Gamma(link="log"), data=d)

##                 prior     class         coef group resp dpar nlpar bound
## 1                             b
## 2                             b Amplitude.st
## 3                             b   DirectionL
## 4 student_t(3, 2, 10) Intercept
## 5 student_t(3, 0, 10)        sd
## 6                            sd                 Id
## 7                            sd    Intercept    Id
## 8   gamma(0.01, 0.01)     shape
```

4.3 Models Estimation

Each model was estimated using 6 independent chains of 8,000 iterations with a *"warm-up"* period of 2,000 iterations, resulting in 36,000 usable samples.

```
set.seed(46)

m.0<-brm(Error1 ~ Amplitude.st + Direction + (1|Id),family=Gamma(link="log"),
         data = d,chains = 6, iter = 8000, warmup = 2000, seed=46)

m.1<-update(m.0, formula. = ~ . + Age + Perception + Environment,
            newdata =  d, warmup=2000, seed=46)

m.2<-update(m.1, formula. = ~ . + Perception:Environment,
            newdata =  d, warmup=2000, seed=46)

m.3<-update(m.1, formula. = ~ . + Age:Environment,
            newdata =  d, warmup=2000, seed=46)

m.4<-update(m.1, formula. = ~ . + Age:Perception,
            newdata =  d, warmup=2000, seed=46)

m.5<-update(m.0, formula. = ~ . + (Age+Perception+Environment)^2,
            newdata =  d, warmup=2000, seed=46)

m.6<-update(m.0, formula. = ~ . + (Age+Perception+Environment)^3,
            newdata =  d, warmup=2000, seed=46)
```

Convergence was evaluated via visual inspection of the trace plots (i.e. sampling chains) and R-hat diagnostic criteria (Gelman & Rubin, 1992). All tested models showed satisfactory convergence with all R-hat $\leq$ 1.00102, where values close to 1 indicate convergence, and no R-hat exceeding the 1.100 proposed threshold for convergence (Gelman et al., 2013). R-hat values and trace plots are reported separetly for each model in the next sections.

4.3.1 m.0

In 6 the trace plots of the parameters in model m.0 are presented. In 6 R-hat values are reported.

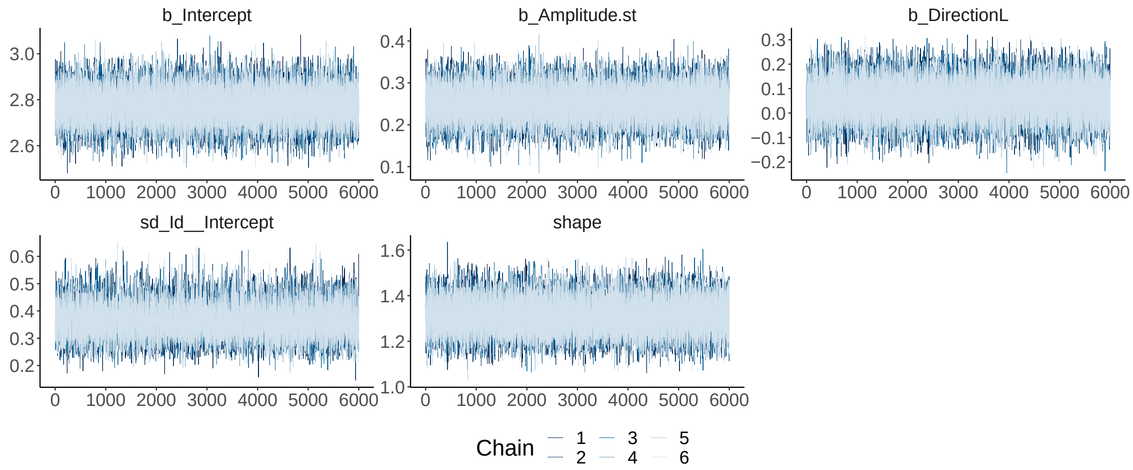

Figure 6: Parameter trace plots m.0. We only represent the iterations after the warm-up.

Table 6: R-hat values model m.0

Parameter	R-hat
**Intercept**	
b_Intercept	1.0000703
**Main Effects**	
b_Amplitude.st	0.9999496
b_DirectionL	0.9999334
**SD and Shape**	
sd_Id__Intercept	1.0002632
shape	0.9998980

4.3.2 `m.1`

In 7 the trace plots of the parameters in model `m.1` are presented. In 7 R-hat values are reported.

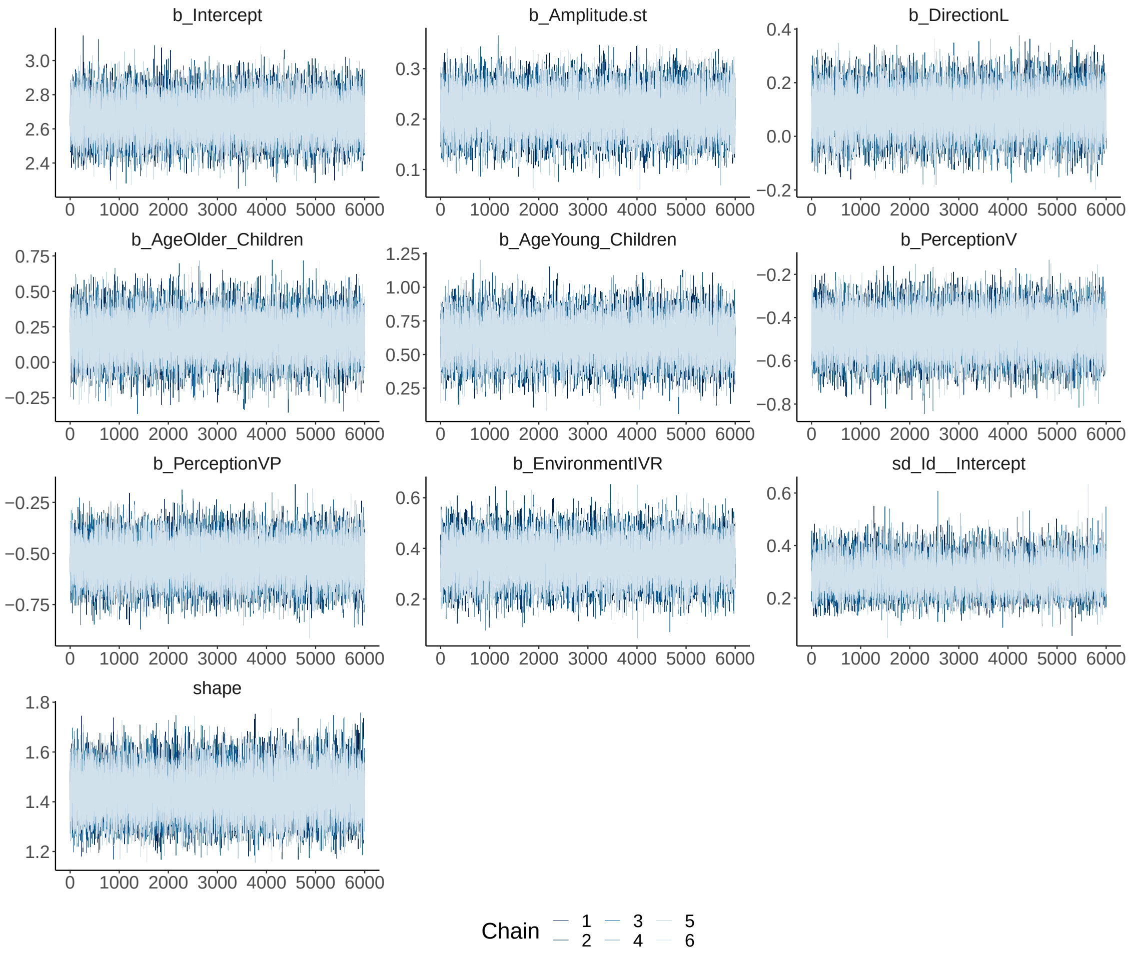

Figure 7: Parameter trace plots `m.1`. We only represent the iterations after the warm-up.

Table 7: R-hat values model `m.1`

Parameter	R-hat
**Intercept**	
b_Intercept	1.0000278
**Main Effects**	
b_Amplitude.st	0.9999213
b_DirectionL	0.9998864
b_AgeOlder_Children	1.0000970
b_AgeYoung_Children	0.9999157
b_PerceptionV	0.9999481
b_PerceptionVP	0.9999765
b_EnvironmentIVR	1.0000876
**SD and Shape**	
sd_Id__Intercept	1.0005022
shape	1.0000356

4.3.3 m.2

In 8 the trace plots of the parameters in model m.2 are presented. In 8 R-hat values are reported.

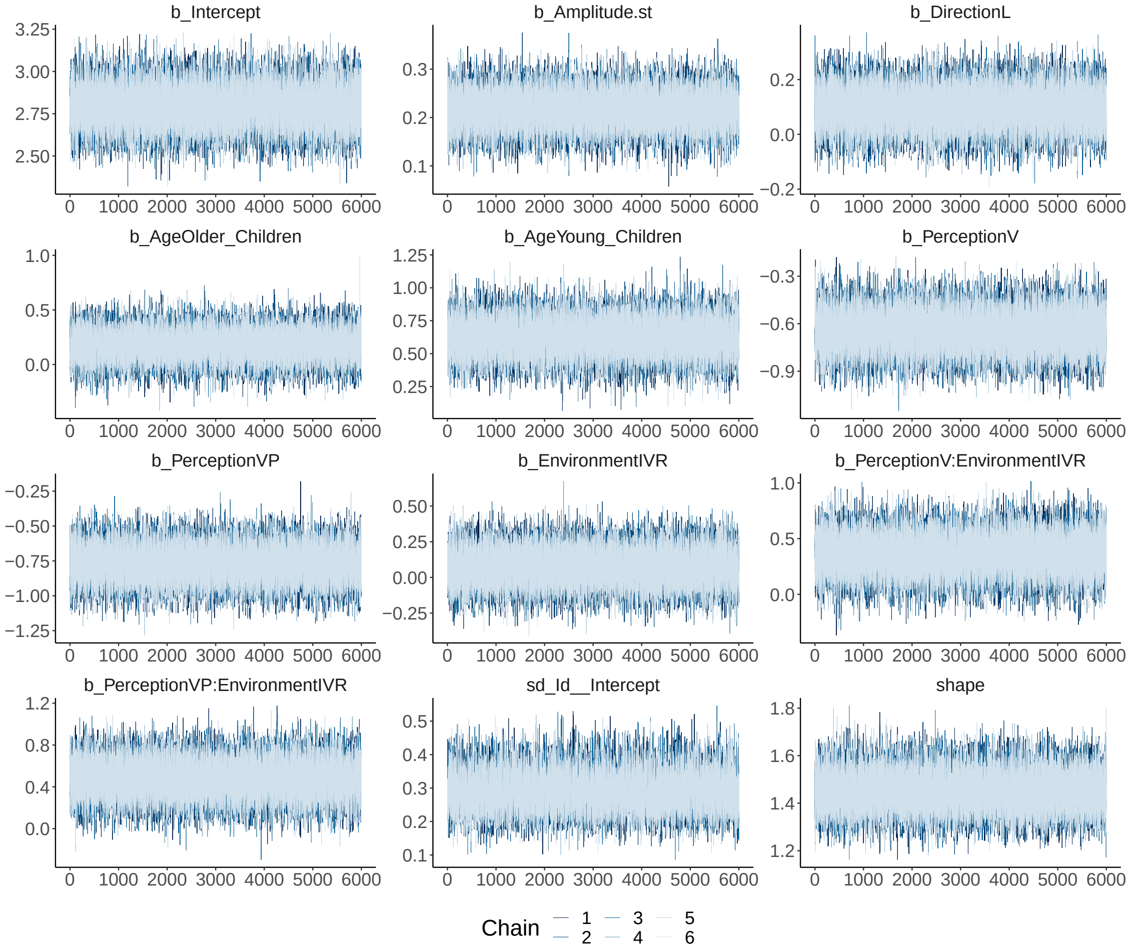

Figure 8: Parameter trace plots m.2. We only represent the iterations after the warm-up.

Table 8: R-hat values model `m.2`

Parameter	R-hat
**Intercept**	
b_Intercept	0.9999611
**Main Effects**	
b_Amplitude.st	0.9999866
b_DirectionL	0.9999106
b_AgeOlder_Children	1.0000100
b_AgeYoung_Children	1.0000832
b_PerceptionV	0.9999024
b_PerceptionVP	0.9999252
b_EnvironmentIVR	0.9999110
**Interaction Effects**	
b_PerceptionV:EnvironmentIVR	1.0000029
b_PerceptionVP:EnvironmentIVR	0.9999393
**SD and Shape**	
sd_Id__Intercept	1.0001767
shape	0.9999588

4.3.4 m.3

In 9 the trace plots of the parameters in model m.3 are presented. In 9 R-hat values are reported.

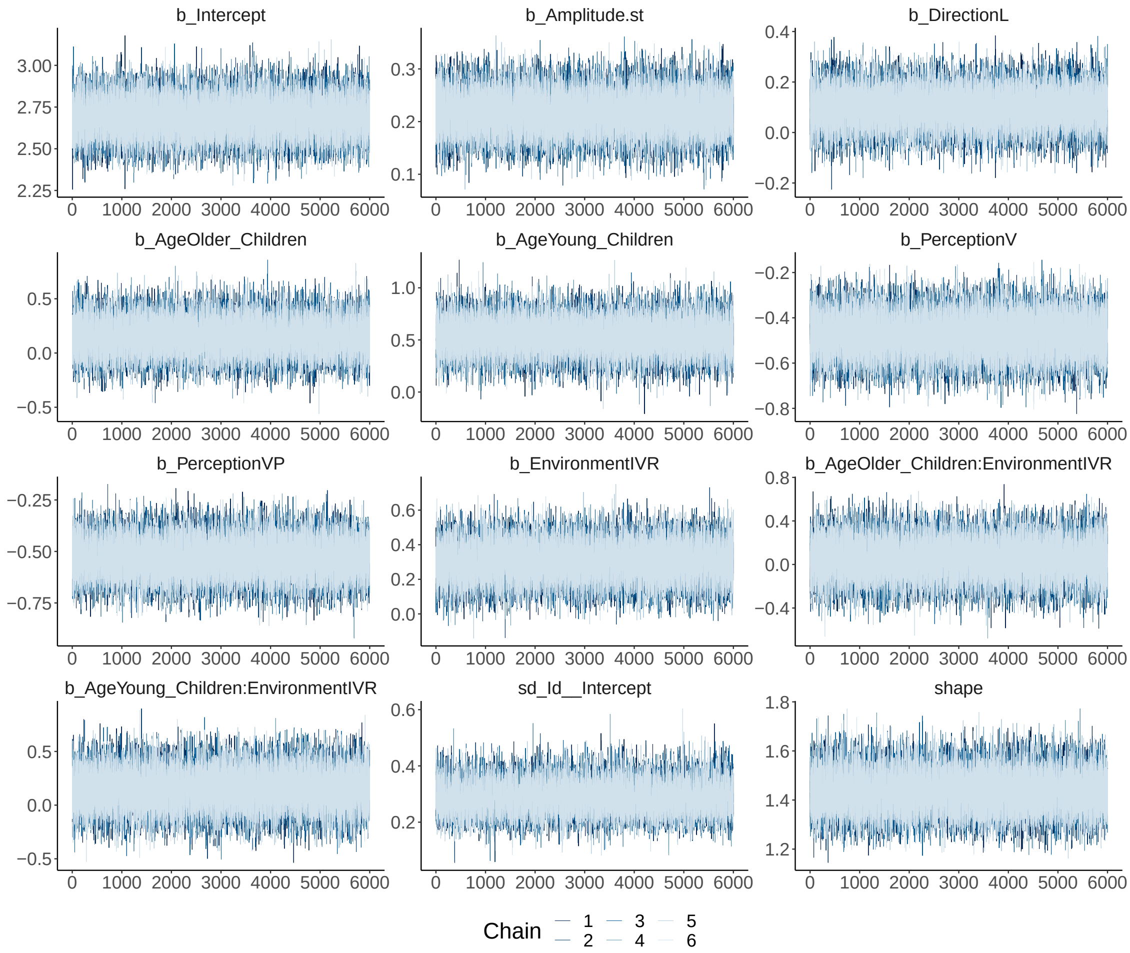

Figure 9: Parameter trace plots m.3. We only represent the iterations after the warm-up.

Table 9: R-hat values model `m.3`

Parameter	R-hat
**Intercept**	
b_Intercept	1.0000509
**Main Effects**	
b_Amplitude.st	0.9998889
b_DirectionL	0.9999307
b_AgeOlder_Children	0.9999849
b_AgeYoung_Children	1.0000911
b_PerceptionV	0.9999024
b_PerceptionVP	0.9998772
b_EnvironmentIVR	0.9999883
**Interaction Effects**	
b_AgeOlder_Children:EnvironmentIVR	0.9999206
b_AgeYoung_Children:EnvironmentIVR	0.9999421
**SD and Shape**	
sd_Id__Intercept	1.0001182
shape	0.9999171

4.3.5 m.4

In 10 the trace plots of the parameters in model m.4 are presented. In 10 R-hat values are reported.

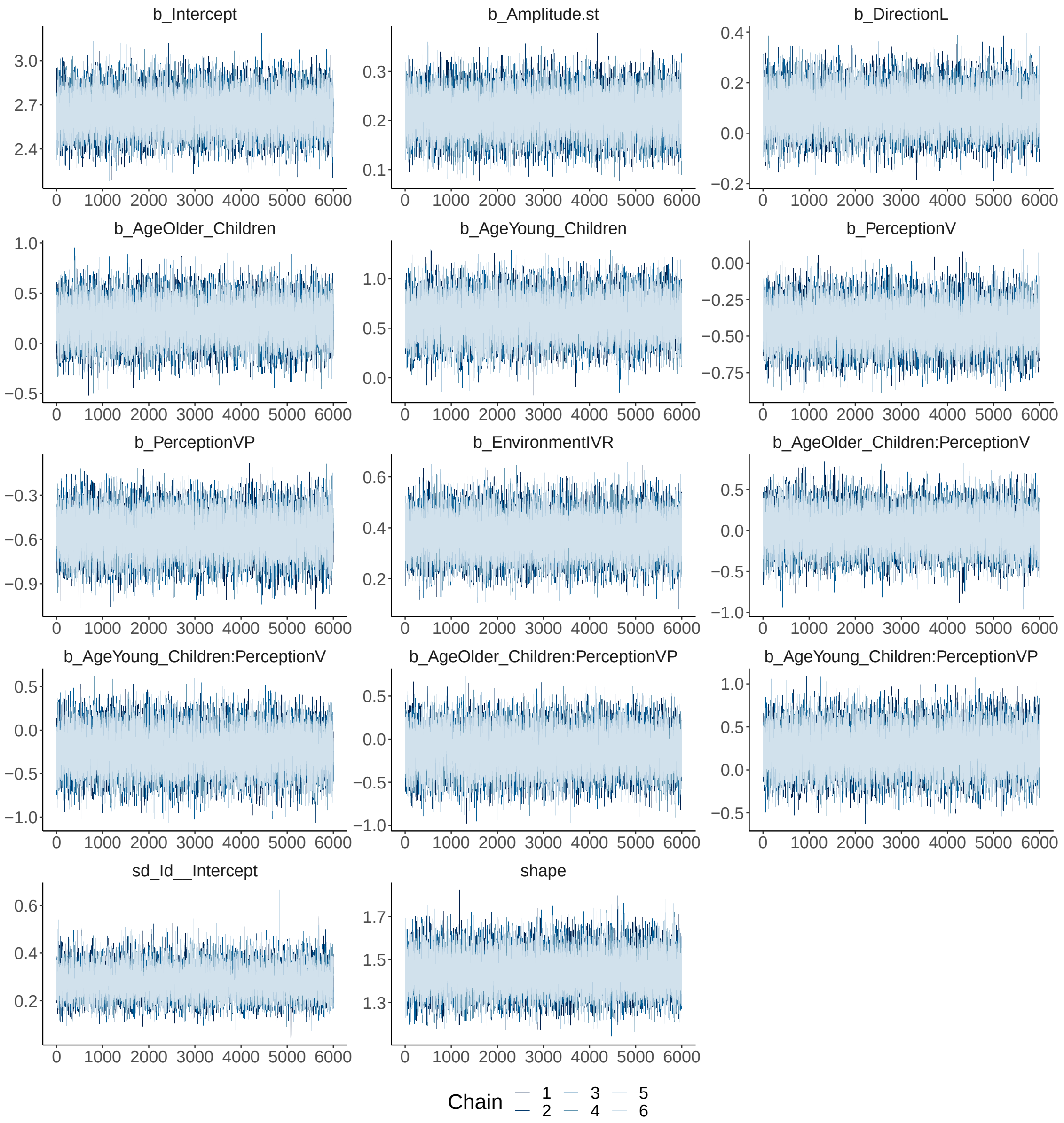

Figure 10: Parameter trace plots m.4. We only represent the iterations after the warm-up.

Table 10: R-hat values model `m.4`

	Parameter	R-hat
**Intercept**		
	b_Intercept	1.0002654
**Main Effects**		
	b_Amplitude.st	0.9999213
	b_DirectionL	0.9999336
	b_AgeOlder_Children	1.0001077
	b_AgeYoung_Children	1.0002796
	b_PerceptionV	0.9999924
	b_PerceptionVP	1.0000110
	b_EnvironmentIVR	0.9999741
**Interaction Effects**		
	b_AgeOlder_Children:PerceptionV	1.0000142
	b_AgeYoung_Children:PerceptionV	0.9999901
	b_AgeOlder_Children:PerceptionVP	1.0000328
	b_AgeYoung_Children:PerceptionVP	1.0000558
**SD and Shape**		
	sd_Id__Intercept	1.0003760
	shape	0.9998865

4.3.6 m.5

In 11 the trace plots of the parameters in model m.5 are presented. In 11 R-hat values are reported.

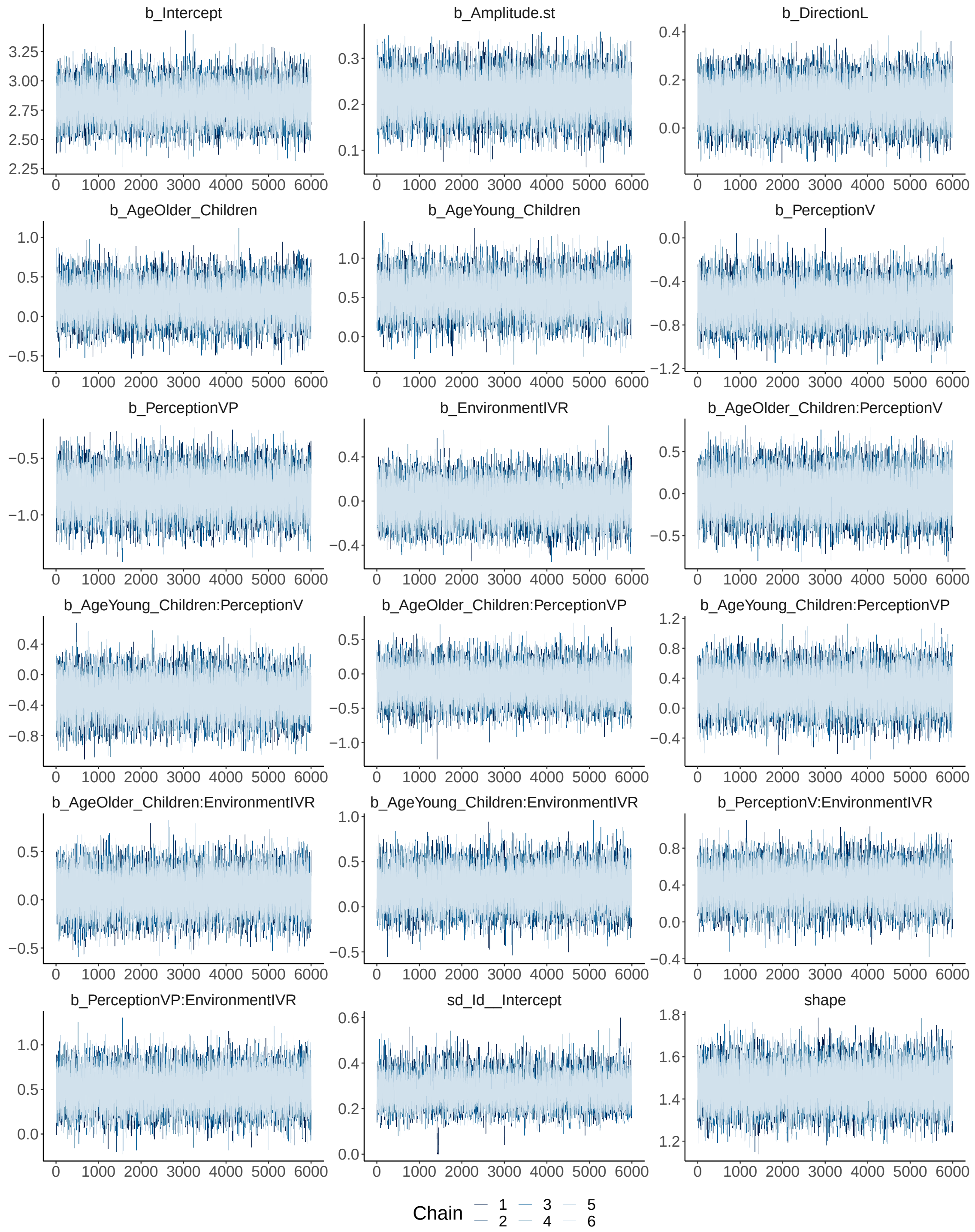

Figure 11: Parameter trace plots m.5. We only represent the iterations after the warm-up.

Table 11: R-hat values model `m.5`

	Parameter	R-hat
**Intercept**		
	b_Intercept	1.0002476
**Main Effects**		
	b_Amplitude.st	0.9999448
	b_DirectionL	1.0000320
	b_AgeOlder_Children	1.0002600
	b_AgeYoung_Children	1.0003221
	b_PerceptionV	1.0000061
	b_PerceptionVP	1.0001615
	b_EnvironmentIVR	1.0000616
**Interaction Effects**		
	b_AgeOlder_Children:PerceptionV	1.0000133
	b_AgeYoung_Children:PerceptionV	1.0000105
	b_AgeOlder_Children:PerceptionVP	1.0000587
	b_AgeYoung_Children:PerceptionVP	1.0001262
	b_AgeOlder_Children:EnvironmentIVR	1.0000978
	b_AgeYoung_Children:EnvironmentIVR	1.0001040
	b_PerceptionV:EnvironmentIVR	0.9999806
	b_PerceptionVP:EnvironmentIVR	1.0000506
**SD and Shape**		
	sd_Id__Intercept	1.0006684
	shape	0.9999478

4.3.7 `m.6`

In 12 the trace plots of the parameters in model `m.6` are presented. In 12 R-hat values are reported.

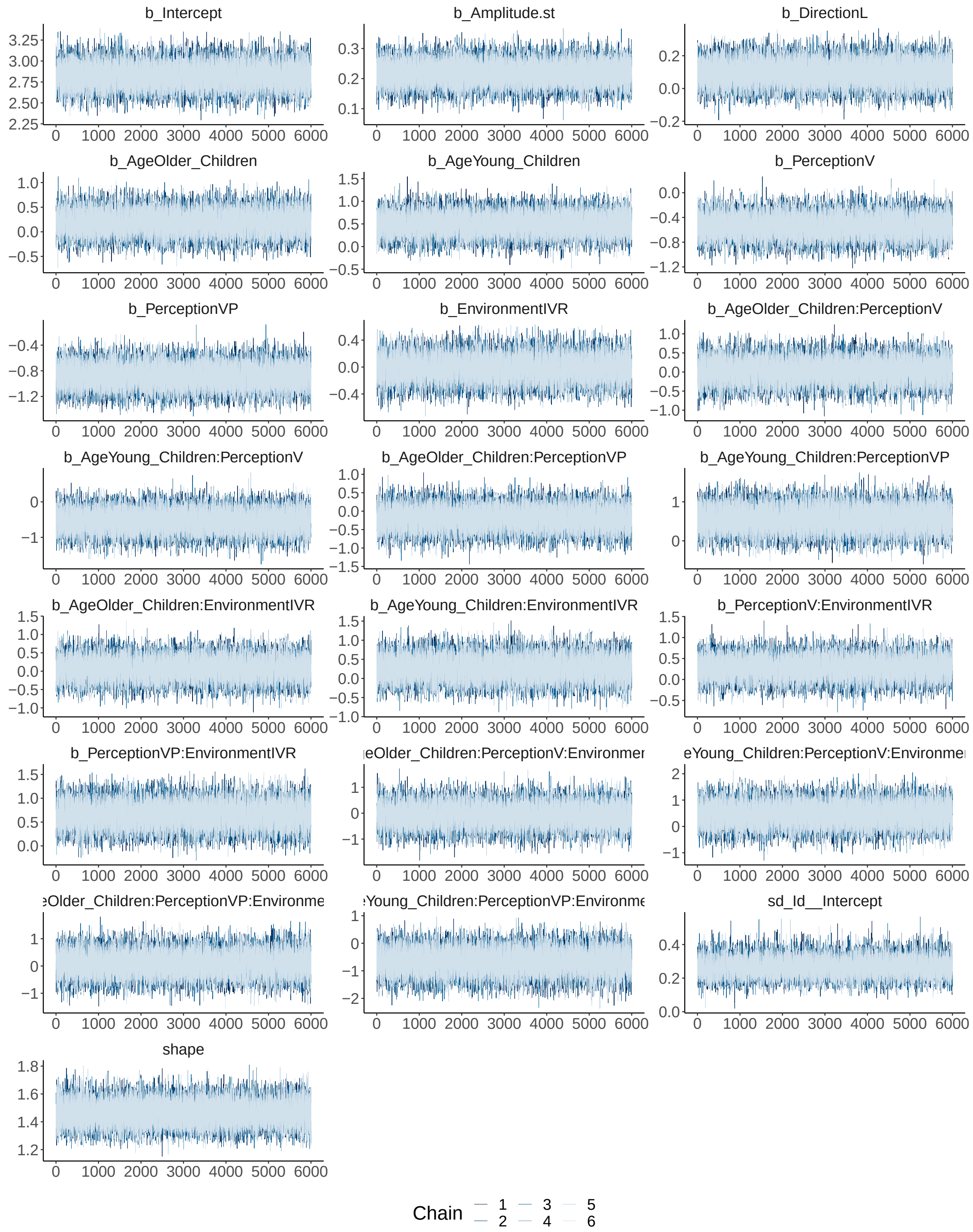

Figure 12: Parameter trace plots `m.6`. We only represent the iterations after the warm-up.

Table 12: R-hat values model `m.6`

	Parameter	R-hat
**Intercept**		
	b_Intercept	1.0002811
**Main Effects**		
	b_Amplitude.st	0.9999474
	b_DirectionL	0.9998861
	b_AgeOlder_Children	1.0003139
	b_AgeYoung_Children	1.0002440
	b_PerceptionV	1.0000941
	b_PerceptionVP	1.0003398
	b_EnvironmentIVR	1.0004737
**Interaction Effects**		
	b_AgeOlder_Children:PerceptionV	1.0001320
	b_AgeYoung_Children:PerceptionV	1.0000189
	b_AgeOlder_Children:PerceptionVP	1.0004903
	b_AgeYoung_Children:PerceptionVP	1.0001956
	b_AgeOlder_Children:EnvironmentIVR	1.0002921
	b_AgeYoung_Children:EnvironmentIVR	1.0002693
	b_PerceptionV:EnvironmentIVR	1.0000812
	b_PerceptionVP:EnvironmentIVR	1.0004311
**Three-ways Interactions**		
	b_AgeOlder_Children:PerceptionV:EnvironmentIVR	1.0001678
	b_AgeYoung_Children:PerceptionV:EnvironmentIVR	1.0000836
	b_AgeOlder_Children:PerceptionVP:EnvironmentIVR	1.0003083
	b_AgeYoung_Children:PerceptionVP:EnvironmentIVR	1.0001604
**SD and Shape**		
	sd_Id__Intercept	0.9999680
	shape	0.9998776

4.4 Model Comparison

The Watanabe-Akaike Information Criterion (WAIC; Gelman, Hwang, & Vehtari, 2014; Vehtari, Gelman, & Gabry, 2017) is used as information criteria to select the most plausible model among the tested models, given the data. WAIC is the corresponding Bayesian version of the commonly used Akaike information criterion (AIC; Akaike, 1973). In line with the Bayesian approach, WAIC has the desirable property of averaging over the posterior distribution rather than conditioning on a point estimate.

Moreover, WAIC-weights for the set of models are computed to present the probability of each model to make the best predictions on new data, conditional on the set of models considered (McElreath, 2016). With this method models are compared using a continuous informative measure of evidence, rather than a dichotomous decision, to avoid premature conclusions in this explorative scenario.

First we compute the WAIC for each model.

```
m.0<-add_waic(m.0)
m.1<-add_waic(m.1)
...
m.6<-add_waic(m.6)
```

Then we compute the relative WAIC weights.

```
WAIC<-data.frame(Model= c("m.0", "m.1","m.2", "m.3","m.4","m.5","m.6"),
                 Waic=c(m.0$waic$estimates[3,1], ... , m.6$waic$estimates[3,1]),
                 SE=c(m.0$waic$estimates[3,2], ... , m.6$waic$estimates[3,2]))
WAIC$rl<- exp((max(WAIC$Waic)-WAIC$Waic)/2) # Relative likelihood of each model
                                            # compared to the worst model
WAIC$w<- round(WAIC$rl/sum(WAIC$rl),2)      # Relative weights
```

WAIC values and WAIC weights are reported in 13.

Table 13: WAIC model comparison

	**Model**	**WAIC**	**SE**	**WAIC weight**
Baseline	`m.0`	4409.8	54.5	0.00
Additive	`m.1`	4349.6	61.9	0.08
2-way Interactions	`m.2`	4345.3	63.2	0.67
	`m.3`	4354.3	62.5	0.01
	`m.4`	4351.3	61.3	0.03
All 2-way Interactions	`m.5`	4349.6	62.7	0.08
3-way Interactions	`m.6`	4348.6	60.9	0.13

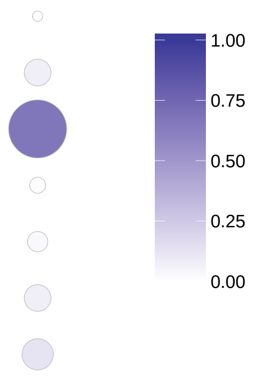

Note: $n_{subjects} = 49; n_{observations} = 578$

Results indicate that `m.2` is the most plausible model that has generated the observed data, having the lower WAIC value (WAIC = 4345.3) and a probability of being the best of 0.67.

Compared with `m.6`, the second-most plausible model with a probability of 0.13, `m.2` is 5.14 times more probable.

5 Description of Model `m.2`

In this section we will first report the estimated parameter of the model `m.2`. Main effects and interaction effects are considered using planned comparison and graphical representations of the predicted values by the model. Moreover, effects sizes are presented. Finally, we will evaluate the model fit considering R^2 value and model Posterior Predictive check.

5.1 Model Parameters

We recall that model `m.2` considers the fixed effects of **Age**, **Amplitude.st**, **Direction**, the interaction between **Perception** and **Environment**, and the random-effect of **Id**.

In 14 95% Bayesian Credible Intervals (BCIs) of the parameter posterior distributions are presented. 95% BCIs represent the range of the 95% most credible parameter values given the prior distribution and the observed data.

Table 14: Estimated parameters of model `m.2`

	Parameters			95 % BCI		Eff.Sample	
	**Name**	**Estimate**	**Est.Error**	**Lower**	**Upper**	**Bulk**	**Tail**
**Random Effects**							
SD	ID Intercept	0.29	0.05	0.19	0.41	15425	23447
**Fixed Effect**							
	Intercept	2.79	0.12	2.57	3.03	30749	29866
	Amplitude.st	0.22	0.04	0.14	0.29	76919	28513
	Direction (left)	0.10	0.07	-0.04	0.23	79506	27632
	Environment (IVR)	0.08	0.12	-0.16	0.32	29652	28643
Age	Older Children	0.18	0.13	-0.09	0.44	28789	27196
	Young Children	0.62	0.13	0.36	0.89	29571	28560
Perception	Vision	-0.64	0.12	-0.89	-0.40	35596	30037
	Vision + Proprioception	-0.77	0.12	-1.01	-0.53	34851	30305
Interaction	Vision : IVR	0.36	0.17	0.02	0.70	32305	29312
	Vision + Proprioception : IVR	0.48	0.17	0.14	0.82	31344	29433
**Family Specific Parameter**							
	shape	1.45	0.08	1.30	1.61	64978	26539

Note: Baseline category for Direction is "Right". Baseline category for Age is "Adults". Baseline category for Perception is "Proprioception". Baseline category for Environment is "Reality". IVR = Immersive Virtual Reality. $n_{subjects} = 49; n_{observations} = 578$

Given the results, Self-turn error is moderated by **Amplitude.st**, **Age** and by the interaction between **Perception** and **Environment**. On the contrary, the **Direction** of rotations seems to have no effect on the subjects' performance ($\beta = 0.1;\ 95\% BCI = -0.04; 0.23$).

In order to interpret the results, we used the posterior parameter distributions to compute, in the original scale, the predicted distribution of Self-turn error means for the condition we are interested in, marginalizing for the other variables.

For example, to obtain the distribution of Self-turn error means for Adults in the Proprioception and Reality condition, which are the baseline model categories, we marginalize (i.e. compute the average score) over the variable direction.

```r
post<-posterior_samples(m.2)  # Obtain the parameters posteriors

adult.proprioception.reality<-(exp(post$b_Intercept)+
                                exp(post$b_Intercept+post$b_DirectionL))/2-1

# We compute the exp to reverse the link function
# Then we calculate the mean

# Remember to subtruct 1 to have the result in the original scale
# (Previously we added 1 for the logarithmic link function)
```

5.1.1 Amplitude

Self-turn error is moderated by **Amplitude.st** ($\beta = 0.22$; $95\% BCI = 0.14; 0.29$), for which increasing levels of task difficulty are associated with a worse performance (13).

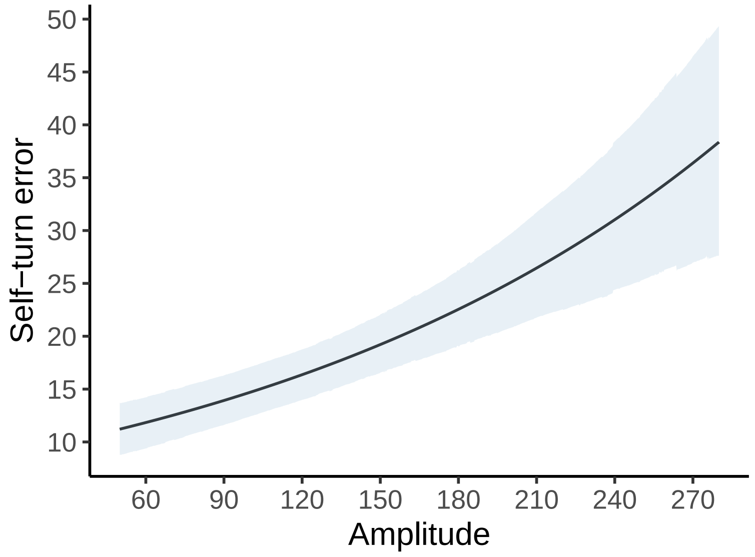

Figure 13: Predicted mean of Self-turn error according to Amplitude.st ($n_{subjects} = 49$; $n_{observations} = 578$). The line represents the mean value. The shaded area represents the 95% BCI values.

5.1.2 Age

To evaluate the role of **Age**, the distributions of predicted mean values for the three groups are presented in 14.

The predicted mean error for Adults is 12.8 degrees ($95\% BCI = 10.6; 15.1$), for Older Children 15.5 degrees ($95\% BCI = 12.1; 19.2$) and for Young Children is 24.8 degrees ($95\% BCI = 19.3; 30.8$). Values are reported in 15 together with Bayesian pairwise comparisons (i.e. predicted score differences between groups).

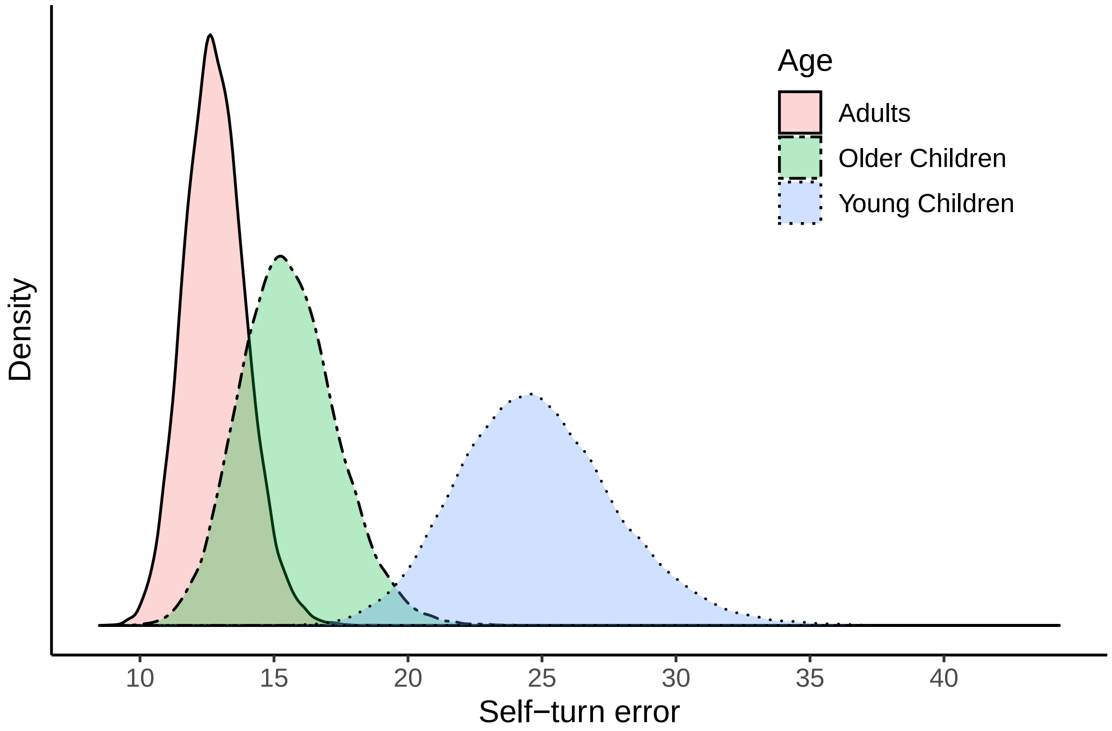

Figure 14: Distributions of the predicted means of Self-turn error according to Age ($n_{subjects} = 49$; $n_{observations} = 578$).

Overall, Young Children are expected to make more Self-turn error than Adults ($95\% BCI = 6.3; 18.2$) and also more than Older Children ($95\% BCI = 2.8; 16$). On the contrary, we cannot state that Older Children are expected to make more Self-turn error because the 95% BCI of the difference includes the value zero ($95\% BCI = -1.4; 6.9$).

Table 15: Predicted means and differences of Self-turn error according to age.

**Mean**		**95% BCI**	
	**Estimate**	**Lower**	**Upper**
**Group**			
Adults	12.8	10.6	15.1
Older Children	15.5	12.1	19.2
Young Children	24.8	19.3	30.8
**Comparisons**			
Young Children - Adults	12.0	6.3	18.2
Young - Older Children	9.3	2.8	16.0
Older Children - Adults	2.7	-1.4	6.9

Note: $n_{subjects} = 49; n_{observations} = 578$

5.1.3 Interaction between Perception and Environment

To interpret the interaction between Perception and Environment conditions, the distributions of predicted mean values for all six conditions are presented in 15.

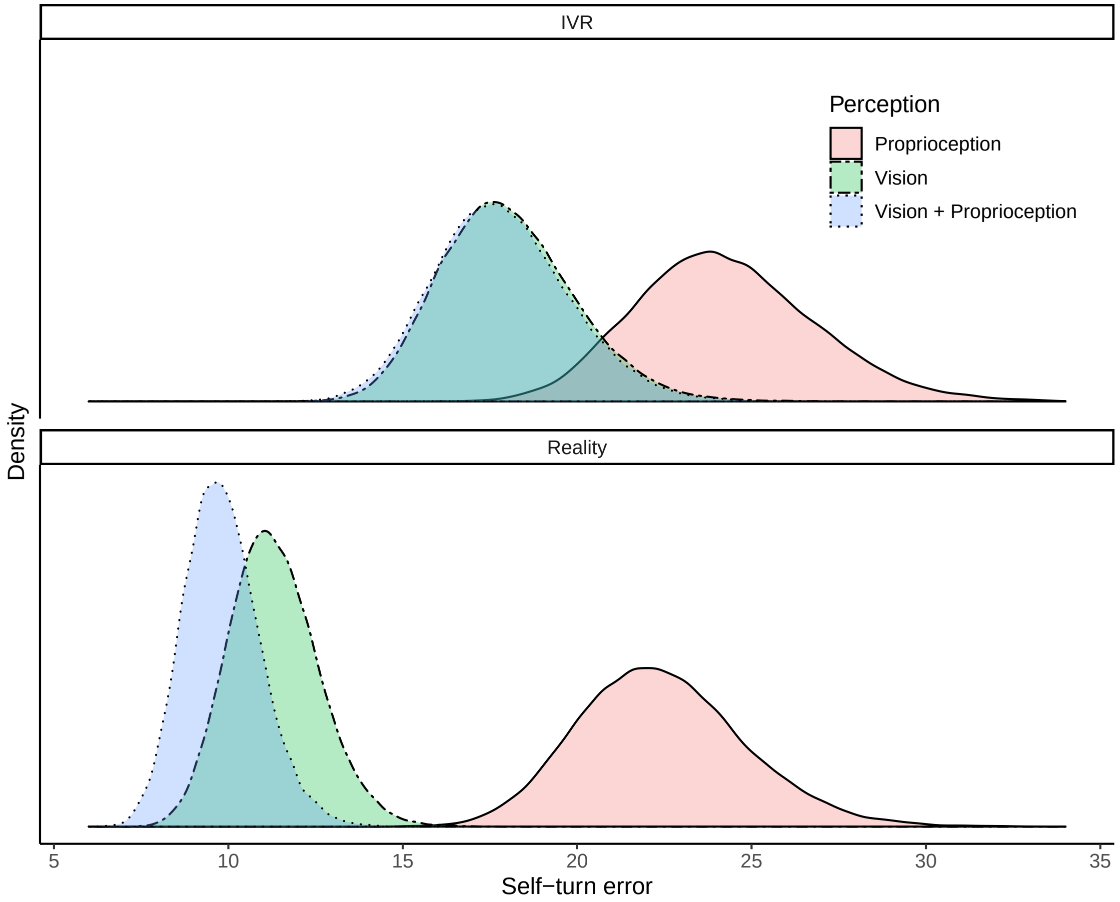

Figure 15: Distributions of the predicted means of Self-turn error according to the experimental conditions ($n_{subjects} = 49$; $n_{observations} = 578$).

In the Reality Environment, the predicted mean error for Proprioception is 22.4 degrees ($95\%BCI = 18; 27.2$), for Vision 11.3 degrees ($95\%BCI = 9; 13.9$) and for Vision + Proprioception is 9.8 degrees ($95\%BCI = 7.8; 12$). In the IVR Environment, the predicted mean error for Proprioception is 24.3 degrees ($95\%BCI = 19.3; 29.2$), for Vision 18 degrees ($95\%BCI = 14.4; 21.8$) and for Vision + Proprioception is 17.8 degrees ($95\%BCI = 14.2; 21.7$). Values are reported in 16 together with Bayesian pairwise comparisons (i.e. predicted score differences between conditions).

Table 16: Predicted means and differences of Self-turn error according experimental conditions.

		Mean	95 % BCI	
		**Estimate**	**Lower**	**Upper**
**Conditions**				
Reality	Proprioception	22.4	18.0	27.2
	Vision	11.3	9.0	13.9
	Vision + Proprioception	9.8	7.8	12.0
IVR	Proprioception	24.3	19.3	29.2
	Vision	18.0	14.4	21.8
	Vision + Proprioception	17.8	14.2	21.7
**Comparisons**				
Reality	Proprioception - Vision	11.1	6.5	15.8
	Proprioception - Vision + Proprioception	12.6	8.0	17.2
	Vision - Vision + Proprioception	1.5	-1.4	4.4
IVR	Proprioception - Vision	6.2	0.9	11.7
	Proprioception - Vision + Proprioception	6.4	0.8	11.7
	Vision - Vision + Proprioception	0.2	-4.3	4.9
IVR - Reality	Proprioception	1.8	-4.3	7.5
	Vision	6.7	2.8	10.6
	Vision + Proprioception	8.0	4.3	11.9

Note: IVR = Immersive Virtual Reality. $n_{subjects} = 49; n_{observations} = 578$

Results showed that, in Reality and IVR Environment, subjects are expected to make more Self-turn error when they rely only on Proprioception than when they can use only Vision (Reality: $95\%BCI = 6.5; 15.8$; IVR: $95\%BCI = 0.9; 11.7$) or Vision + Proprioception (Reality: $95\%BCI = 8; 17.2$; IVR: $95\%BCI = 0.8; 11.7$). On the contrary, there is no difference between the use of Vision and Vision + Proprioception (Reality: $95\%BCI = -1.4; 4.4$; IVR: $95\%BCI = -4.3; 4.9$). Moreover, compariong IVR to Reality Environment, Self-turn errors increase when subjects rely only on Vision ($95\%BCI = 2.8; 10.6$) or on Vision + Proprioception ($95\%BCI = 4.3; 11.9$), but subjects are not expected to make the more errors when they rely only on Proprioception ($95\%BCI = -4.3; 7.5$).

5.2 Effect Sizes

To quantify the differences between the various age groups or the different conditions, we expressed the effects as the ratio between the two scores of the comparison of interest (Table 17).

Table 17: Effect size as the ratio of the scores of the different Age groups or experimental conditions.

		Effect Size	95 % BCI	
		**Estimate**	**Lower**	**Upper**
**Age**				
	Young Children / Adults	1.88	1.39	2.37
	Young / Older Children	1.58	1.12	2.06
	Older Children / Adults	1.20	0.90	1.53
**Conditions**				
Reality	Proprioception / Vision	1.92	1.48	2.41
	Proprioception / Vision + Proprioception	2.18	1.68	2.73
	Vision / Vision + Proprioception	1.15	0.87	1.43
IVR	Proprioception / Vision	1.34	1.03	1.66
	Proprioception / Vision + Proprioception	1.35	1.03	1.68
	Vision / Vision + Proprioception	1.02	0.78	1.26
IVR / Reality	Proprioception	1.09	0.83	1.34
	Vision	1.56	1.19	1.94
	Vision + Proprioception	1.75	1.34	2.18

Note:
IVR = Immersive Virtual Reality. $n_{subjects} = 49; n_{observations} = 578$

For example, Young Children are expected to make 88% more errors than Adults and 58% more errors than Older Children. Considering the Reality Environment, when using only Proprioception subjects are expected to make 92% more errors than when they rely only on Vision and 118% more errors than when using Vision + Proprioception. Considering the IVR Environment, when using only Proprioception subjects are expected to make 34% more errors than when they rely only on Vision and 35% more errors than when using Vision + Proprioception. Moreover, comparing IVR to Reality Environment, subjects are expected to make 56% more errors when using only Vision and 75% more when using Vision + Proprioception.

5.3 Model Fit

Commonly, the model fit (i.e. the model's ability to explain the data) is assessed using statistical indexes as R^2 (Xu, 2003). However, the usual definition of R^2 (i.e. the variance of the predicted values divided by the variance of the data) has some problems in the Bayesian framework because the numerator can be larger than the denominator (Gelman, Goodrich, Gabry, & Vehtari, 2018). To overcome this issue, Gelman et al. (2018) proposed a Bayesian alternative definition of R^2:

$$Bayesian\ R^2 = \frac{Explained\ Variance}{Explained\ Variance + Residual\ Variance} = \frac{V_{n=1}^{N}\hat{y}_n}{V_{n=1}^{N}\hat{y}_n + V_{n=1}^{N}r_n} \quad (1)$$

where $r_n = y_n - \hat{y_n}$ are the residuals of the fitted model. Given this definition, Bayesian R^2 is always between 0 and 1 by construction, and thus it follows the same interpretation as the common R^2: an estimate of the proportion of variance explained.

The estimated value of Bayesian R^2 for the model `m.2` is 0.26 ($95\% BCI = 0.19; 0.34$), that is the model explains 26% of the variability of the data (Table 18).

Table 18: Bayesian R^2

Bayesian R^2		95 % BCI	
**Estimate**	**SE**	**Lower**	**Upper**
0.26	0.038	0.187	0.337

Note: $n_{subjects} = 49; n_{observations} = 578$

Posterior predictive check is an alternative way to evaluate model goodness-of-fit (Kruschke, 2013). Qualitative visual inspection of the posterior predictive check allows us to assess whether data predicted by the model resemble the observed data or if the residuals between actual and simulated data follow certain patterns.

In 16 the data predicted by 100 sampled parameter vectors are presented. Visual inspection reveals a good fit of the model with the predicted data that properly and consistently follow the observed data.

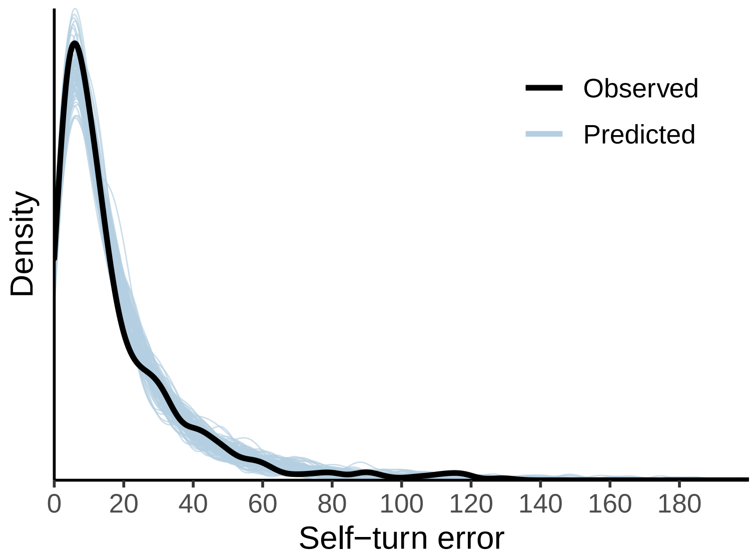

Figure 16: Posterior predictive check. The black line represents the distribution of observed data ($n_{subjects} = 49$; $n_{observations} = 578$). The gray lines represent the distribution of predicted data for each of the sampled parameter vector ($n_{samples} = 100$).

6 Maximum Likelihood

In this section all models are estimated with the traditional maximum likelihood approach using the R package `lme4` (Bates et al., 2014). In several cases, convergence is not reached (i.e. unreliable results).

```
glm.0<-glmer(Error1 ~ Amplitude.st + Direction + (1|Id),
             family=Gamma(link="log"),data = d)
glm.1<-update(glm.0, formula. = ~ . + Age + Perception + Environment)
glm.2<-update(glm.1, formula. = ~ . + Perception:Environment)
glm.3<-update(glm.1, formula. = ~ . + Age:Environment)
glm.4<-update(glm.1, formula. = ~ . + Age:Perception)

## Warning in checkConv(attr(opt, "derivs"), opt$par, ctrl = control$checkConv, :
Model failed to converge with max|grad| = 0.0105459 (tol = 0.001, component 1)

glm.5<-update(glm.0, formula. = ~ . + (Age+Perception+Environment)^2)

## Warning in checkConv(attr(opt, "derivs"), opt$par, ctrl = control$checkConv, :
Model failed to converge with max|grad| = 0.00112266 (tol = 0.001, component 1)

glm.6<-update(glm.0, formula. = ~ . + (Age+Perception+Environment)^3)

## Warning in checkConv(attr(opt, "derivs"), opt$par, ctrl = control$checkConv, :
Model failed to converge with max|grad| = 0.0058201 (tol = 0.001, component 1)
```

In 19 we compare the estimated model parameters using the maximum likelihood approach (`glm.2`) and the Bayesian approach (`m.2`).

Overall, values are very similar.

Table 19: Comparison between Bayesian `m.2` and Maximum-Likelihood `glm.2` models

Parameters	m.2		glm.2	
**Name**	**Estimate**	**Est.Error**	**Estimate**	**Std.Error**
Intercept	2.79	0.12	2.77	0.12
Amplitude.st	0.22	0.04	0.22	0.04
Direction (left)	0.10	0.07	0.09	0.07
Environment (IVR)	0.08	0.12	0.07	0.12
**Age**				
Older Children	0.18	0.13	0.17	0.14
Young Children	0.62	0.13	0.62	0.14
**Perception**				
Vision	-0.64	0.12	-0.65	0.12
Vision + Proprioception	-0.77	0.12	-0.77	0.12
**Interaction**				
Vision : IVR	0.36	0.17	0.37	0.17
Vision + Proprioception : IVR	0.48	0.17	0.48	0.17

Note: Baseline category for Direction is “right”. Baseline category for Age is “Adults”. Baseline category for Perception is “Proprioception”. Baseline category for Environment is “Reality”. IVR = Immersive Virtual Reality. $n_{subjects} = 49$; $n_{observations} = 578$

7 Session Information

```r
sessionInfo(package = NULL)
```

```
## R version 3.6.1 (2019-07-05)
## Platform: x86_64-apple-darwin15.6.0 (64-bit)
## Running under: macOS Mojave 10.14.6
##
## Matrix products: default
## BLAS:   /Library/Frameworks/R.framework/Versions/3.6/Resources/lib/libRblas.0.dylib
## LAPACK: /Library/Frameworks/R.framework/Versions/3.6/Resources/lib/libRlapack.dylib
##
## locale:
## [1] en_US.UTF-8/en_US.UTF-8/en_US.UTF-8/C/en_US.UTF-8/en_US.UTF-8
##
## attached base packages:
## [1] grid      stats     graphics  grDevices utils     datasets  methods
## [8] base
##
## other attached packages:
##  [1] gtable_0.3.0       bayesplot_1.7.1    gridExtra_2.3      ggcorrplot_0.1.3
##  [5] kableExtra_1.1.0   HDInterval_0.2.0   rstan_2.19.2       StanHeaders_2.19.0
##  [9] brms_2.11.0        Rcpp_1.0.3         lme4_1.1-21        Matrix_1.2-17
## [13] ggplot2_3.2.1      knitr_1.26
##
## loaded via a namespace (and not attached):
##  [1] minqa_1.2.4          colorspace_1.4-1     class_7.3-15
##  [4] ggridges_0.5.2       rsconnect_0.8.16     markdown_1.1
##  [7] base64enc_0.1-3      rstudioapi_0.10      farver_2.0.1
## [10] DT_0.11              fansi_0.4.0          xml2_1.2.2
## [13] bridgesampling_0.7-2 codetools_0.2-16     splines_3.6.1
## [16] shinythemes_1.1.2    zeallot_0.1.0        nloptr_1.2.1
## [19] shiny_1.4.0          readr_1.3.1          compiler_3.6.1
## [22] httr_1.4.1           backports_1.1.5      assertthat_0.2.1
## [25] fastmap_1.0.1        lazyeval_0.2.2       cli_2.0.0
## [28] later_1.0.0          htmltools_0.4.0      prettyunits_1.0.2
## [31] tools_3.6.1          igraph_1.2.4.2       coda_0.19-3
## [34] glue_1.3.1           reshape2_1.4.3       dplyr_0.8.3
## [37] vctrs_0.2.1          nlme_3.1-141         crosstalk_1.0.0
## [40] xfun_0.11            stringr_1.4.0        ps_1.3.0
## [43] rvest_0.3.4          mime_0.8             miniUI_0.1.1.1
## [46] lifecycle_0.1.0      gtools_3.8.1         MASS_7.3-51.4
## [49] zoo_1.8-6            scales_1.1.0         colourpicker_1.0
## [52] hms_0.5.2            promises_1.1.0       Brobdingnag_1.2-6
## [55] parallel_3.6.1       inline_0.3.15        shinystan_2.5.0
## [58] loo_2.2.0            stringi_1.4.3        highr_0.8
## [61] dygraphs_1.1.1.6     e1071_1.7-3          boot_1.3-23
## [64] pkgbuild_1.0.6       rlang_0.4.2          pkgconfig_2.0.3
## [67] matrixStats_0.55.0   evaluate_0.14        lattice_0.20-38
## [70] purrr_0.3.3          rstantools_2.0.0     htmlwidgets_1.5.1
## [73] labeling_0.3         processx_3.4.1       tidyselect_0.2.5
## [76] plyr_1.8.5           magrittr_1.5         R6_2.4.1
## [79] pillar_1.4.3         withr_2.1.2          xts_0.11-2
```

```
## [82] abind_1.4-5         tibble_2.1.3        crayon_1.3.4
## [85] rmarkdown_1.16      callr_3.4.0         threejs_0.3.1
## [88] digest_0.6.23       webshot_0.5.1       xtable_1.8-4
## [91] httpuv_1.5.2        stats4_3.6.1        munsell_0.5.0
## [94] viridisLite_0.3.0   shinyjs_1.1
```

References

Akaike, H. (1973). Information theory and an extension of the maximum likelihood principle. In *Second International Symposium on Information Theory* (pp. 267–281). Petrov, B. N. and Csaki, F., editors.

Bates, D., Mächler, M., Bolker, B., & Walker, S. (2014). Fitting Linear Mixed-Effects Models using lme4. *arXiv:1406.5823 [stat]*. arXiv: 1406.5823 [stat]

Bolker, B., Brooks, M., Clark, C., Geange, S., Poulsen, J., Stevens, M., & White, J. (2009). Generalized linear mixed models: A practical guide for ecology and evolution. *Trends in Ecology & Evolution*, *24*(3), 127–135. doi:10.1016/j.tree.2008.10.008

Bürkner, P.-C. (2017). Brms: An R package for Bayesian multilevel models using Stan. *Journal of Statistical Software*, *80*(1), 1–28. doi:10.18637/jss.v080.i01

Etz, A., & Vandekerckhove, J. (2018). Introduction to Bayesian Inference for Psychology. *Psychonomic Bulletin & Review*, *25*(1), 5–34. doi:10.3758/s13423-017-1262-3

Fong, Y., Rue, H., & Wakefield, J. (2010). Bayesian inference for generalized linear mixed models. *Biostatistics*, *11*(3), 397–412. doi:10.1093/biostatistics/kxp053

Fox, J. (2016). *Applied regression analysis and generalized linear models* (Third Edition). OCLC: ocn894301740. Los Angeles: SAGE.

Gelman, A., Carlin, J. B., Stern, H. S., Dunson, D. B., Vehtari, A., & Rubin, D. B. (2013). *Bayesian data analysis.* doi:10.1201/b16018

Gelman, A., Goodrich, B., Gabry, J., & Vehtari, A. (2018). R-squared for Bayesian regression models. *The American Statistician*, 1–6. doi:10.1080/00031305.2018.1549100

Gelman, A., Hwang, J., & Vehtari, A. (2014). Understanding predictive information criteria for Bayesian models. *Statistics and Computing*, *24*(6), 997–1016. doi:10.1007/s11222-013-9416-2

Gelman, A., & Rubin, D. B. (1992). Inference from iterative simulation using multiple sequences. *Statistical Science*, *7*(4), 457–472.

Hoffman, M. D., & Gelman, A. (2014). The No-U-Turn Sampler: Adaptively Setting Path Lengths in Hamiltonian Monte Carlo. *Journal of Machine Learning Research*, *15*, 1593–1623. arXiv: 1111.4246

Kruschke, J. K. (2013). Posterior predictive checks can and should be Bayesian: Comment on Gelman and Shalizi, 'Philosophy and the practice of Bayesian statistics'. *British Journal of Mathematical and Statistical Psychology*, *66*(1), 45–56. doi:10.1111/j.2044-8317.2012.02063.x

Kruschke, J. K., & Liddell, T. M. (2018). The Bayesian new statistics: Hypothesis testing, estimation, meta-analysis, and power analysis from a Bayesian perspective. *Psychonomic Bulletin & Review*, *25*(1), 178–206. doi:10.3758/s13423-016-1221-4

Lo, S., & Andrews, S. (2015). To transform or not to transform: Using generalized linear mixed models to analyse reaction time data. *Frontiers in Psychology*, *6*. doi:10.3389/fpsyg.2015.01171

McElreath, R. (2016). *Statistical Rethinking: A Bayesian Course with Examples in R and Stan* (1st ed.). doi:10.1201/9781315372495

Neal, R. (2012). MCMC using Hamiltonian dynamics. In S. Brooks, A. Gelman, G. Jones, & X. Meng (Eds.), *Handbook of Markov chain Monte Carlo* (pp. 113–162). http://arxiv.org/abs/1206.1901. New York, NY: Chapman.

Ng, V. K., & Cribbie, R. A. (2017). Using the Gamma Generalized Linear Model for Modeling Continuous, Skewed and Heteroscedastic Outcomes in Psychology. *Current Psychology*, *36*(2), 225–235. doi:10.1007/s12144-015-9404-0

R Core Team. (2018). R: A language and environment for statistical computing. R Foundation for Statistical Computing. https://www.R-project.org/. Vienna, Austria.

Romeijn, J., & van de Schoot, R. [R.]. (2008). A philosopher's view on Bayesian evaluation of informative hypotheses. In H. Hoijtink, I. Klugkist, & P. Boelen (Eds.), *Bayesian evaluation of informative hypotheses* (pp. 329–357). doi:10.1007/978-0-387-09612-4_16

Stan Development Team. (2017a). Stan Modeling Language: User's Guide and Reference Manual. http://mc-stan.org/manual.html.

Stan Development Team. (2017b). Stan: A C++ library for probability and sampling. http://mc-stan. org.

van de Schoot, R. [Rens], Kaplan, D., Denissen, J., Asendorpf, J. B., Neyer, F. J., & van Aken, M. A. (2014). A gentle introduction to Bayesian analysis: Applications to developmental research. *Child Development*, *85*(3), 842–860. doi:10.1111/cdev.12169

Vandekerckhove, J., Matzke, D., & Wagenmakers, E.-J. (2015). *Model Comparison and the Principle of Parsimony* (J. R. Busemeyer, Z. Wang, J. T. Townsend, & A. Eidels, Eds.). Oxford University Press.

Vehtari, A., Gelman, A., & Gabry, J. (2017). Practical Bayesian model evaluation using leave-one-out cross-validation and WAIC. *Statistics and Computing*, *27*(5), 1413–1432. doi:10.1007/s11222-016-9696-4

Wagenmakers, E.-J., & Farrell, S. (2004). AIC model selection using Akaike weights. *Psychonomic Bulletin & Review*, *11*(1), 192–196. doi:10.3758/BF03206482

Xu, R. (2003). Measuring explained variation in linear mixed effects models. *Statistics in Medicine*, (22), 3527–3541. doi:10.1002/sim.1572
