# Supplementary material for: Proprioceptive accuracy in Immersive Virtual Reality: A developmental perspective
Source: PLoS One. 2020 Jan 30;15(1):e0222253. doi: 10.1371/journal.pone.0222253 (PMC6992210; doi:10.1371/journal.pone.0222253)
Supplement: S1 Table — (PDF) [file pone.0222253.s002.pdf]

S1 Table. Number of observations according to Age groups and conditions.

	Proprioception	Vision	Vision + Proprioception	Total
**Reality**				
Adults	46	45	46	137
Older Children	26	26	25	77
Young Children	25	25	25	75
Total	97	96	96	289
**IVR**				
Adults	44	48	46	138
Older Children	26	26	26	78
Young Children	24	25	24	73
Total	94	99	96	289
**Total**				
Adults	90	93	92	275
Older Children	52	52	51	155
Young Children	49	50	49	148
Total	191	195	192	578

Note: IVR = Immersive Virtual Reality. $n_{subjects} = 49; n_{observations} = 578$
