# Supplementary material for: Proprioceptive accuracy in Immersive Virtual Reality: A developmental perspective
Source: PLoS One. 2020 Jan 30;15(1):e0222253. doi: 10.1371/journal.pone.0222253 (PMC6992210; doi:10.1371/journal.pone.0222253)
Supplement: S2 Table — (PDF) [file pone.0222253.s003.pdf]

**S2 Table.** Model formulas

| | | | | Predictors | |
|---|---|---|---|---|---|
| | **Model** | **Dependent Variable** | | **Fixed Effects** | **Random Effect** |
| Baseline | `m.0` | error | $\leftarrow$ | Amplitude.st + Direction | Id |
| Additive | `m.1` | error | $\leftarrow$ | m.0 + Age + Perception + Environment | Id |
| 2-way Interactions | `m.2` | error | $\leftarrow$ | m.1 + Perception $\times$ Environment | Id |
| | `m.3` | error | $\leftarrow$ | m.1 + Age $\times$ Environment | Id |
| | `m.4` | error | $\leftarrow$ | m.1 + Age $\times$ Perception | Id |
| All 2-way Interactions | `m.5` | error | $\leftarrow$ | m.1 + Perception $\times$ Environment+ Age $\times$ Environment + Age $\times$ Perception | Id |
| 3-way Interactions | `m.6` | error | $\leftarrow$ | m.5 + Age $\times$ Perception $\times$ Environment | Id |
