# Supplementary material for: Proprioceptive accuracy in Immersive Virtual Reality: A developmental perspective
Source: PLoS One. 2020 Jan 30;15(1):e0222253. doi: 10.1371/journal.pone.0222253 (PMC6992210; doi:10.1371/journal.pone.0222253)
Supplement: S3 Table — (PDF) [file pone.0222253.s004.pdf]

S3 Table. WAIC model comparison

	Model	WAIC	SE	WAIC weight
Baseline	`m.0`	4409.8	54.5	0.00
Additive	`m.1`	4349.6	61.9	0.08
2-way Interactions	`m.2`	4345.3	63.2	0.67
	`m.3`	4354.3	62.5	0.01
	`m.4`	4351.3	61.3	0.03
All 2-way Interactions	`m.5`	4349.6	62.7	0.08
3-way Interactions	`m.6`	4348.6	60.9	0.13

Note: $n_{subjects} = 49; n_{observations} = 578$
