# Supplementary material for: Proprioceptive accuracy in Immersive Virtual Reality: A developmental perspective
Source: PLoS One. 2020 Jan 30;15(1):e0222253. doi: 10.1371/journal.pone.0222253 (PMC6992210; doi:10.1371/journal.pone.0222253)
Supplement: S4 Table — (PDF) [file pone.0222253.s005.pdf]

S4 Table. Predicted means and differences of Self-turn error according to age.

Mean		95% BCI	
	**Estimate**	**Lower**	**Upper**
**Group**			
Adults	12.8	10.6	15.1
Older Children	15.5	12.1	19.2
Young Children	24.8	19.3	30.8
**Comparisons**			
Young Children - Adults	12.0	6.3	18.2
Young - Older Children	9.3	2.8	16.0
Older Children - Adults	2.7	-1.4	6.9

Note: $n_{subjects} = 49; n_{observations} = 578$
