# Supplementary material for: Proprioceptive accuracy in Immersive Virtual Reality: A developmental perspective
Source: PLoS One. 2020 Jan 30;15(1):e0222253. doi: 10.1371/journal.pone.0222253 (PMC6992210; doi:10.1371/journal.pone.0222253)
Supplement: S5 Table — (PDF) [file pone.0222253.s006.pdf]

S5 Table. Predicted means and differences of Self-turn error according experimental conditions.

Mean			95 % BCI	
		**Estimate**	**Lower**	**Upper**
**Conditions**				
Reality				
	Proprioception	22.4	18.0	27.2
	Vision	11.3	9.0	13.9
	Vision + Proprioception	9.8	7.8	12.0
IVR				
	Proprioception	24.3	19.3	29.2
	Vision	18.0	14.4	21.8
	Vision + Proprioception	17.8	14.2	21.7
**Comparisons**				
Reality				
	Proprioception - Vision	11.1	6.5	15.8
	Proprioception - Vision + Proprioception	12.6	8.0	17.2
	Vision - Vision + Proprioception	1.5	-1.4	4.4
IVR				
	Proprioception - Vision	6.2	0.9	11.7
	Proprioception - Vision + Proprioception	6.4	0.8	11.7
	Vision - Vision + Proprioception	0.2	-4.3	4.9
IVR - Reality				
	Proprioception	1.8	-4.3	7.5
	Vision	6.7	2.8	10.6
	Vision + Proprioception	8.0	4.3	11.9

Note: IVR = Immersive Virtual Reality. $n_{subjects} = 49; n_{observations} = 578$
