# Supplementary material for: Proprioceptive accuracy in Immersive Virtual Reality: A developmental perspective
Source: PLoS One. 2020 Jan 30;15(1):e0222253. doi: 10.1371/journal.pone.0222253 (PMC6992210; doi:10.1371/journal.pone.0222253)
Supplement: S6 Table — (PDF) [file pone.0222253.s007.pdf]

S6 Table. Effect size as the ratio of the scores of the different Age groups or experimental conditions.

**Effect Size**			**95 % BCI**	
		**Estimate**	**Lower**	**Upper**
**Age**				
	Young Children / Adults	1.88	1.39	2.37
	Young / Older Children	1.58	1.12	2.06
	Older Children / Adults	1.20	0.90	1.53
**Conditions**				
Reality				
	Proprioception / Vision	1.92	1.48	2.41
	Proprioception / Vision + Proprioception	2.18	1.68	2.73
	Vision / Vision + Proprioception	1.15	0.87	1.43
IVR				
	Proprioception / Vision	1.34	1.03	1.66
	Proprioception / Vision + Proprioception	1.35	1.03	1.68
	Vision / Vision + Proprioception	1.02	0.78	1.26
IVR/Reality				
	Proprioception	1.09	0.83	1.34
	Vision	1.56	1.19	1.94
	Vision + Proprioception	1.75	1.34	2.18

Note: IVR = Immersive Virtual Reality. $n_{subjects} = 49; n_{observations} = 578$
