# Supplementary material for: Proprioceptive accuracy in Immersive Virtual Reality: A developmental perspective
Source: PLoS One. 2020 Jan 30;15(1):e0222253. doi: 10.1371/journal.pone.0222253 (PMC6992210; doi:10.1371/journal.pone.0222253)
Supplement: Attachment — Submitted filename: PLOSONE_VRFarroni.docx [file pone.0222253.s008.docx]

MAJOR POINTS

- In general the ms is very long, and would benefit from some editing. E.g. way too much detail on ordering conditions.
- In the intro, I appreciate your attempt to carefully compare the senses and have no problem with you defining proprioception as the perception of the body posture, rather than as information which comes through particular sensory channels (from muscles & joints). But you are not consistent about it. Thus we have “proprioception belongs to the somatosensory system” (channel-specific); followed by discussion of visuo-proprioceptive info (focussed on the object of perception); and then back to “proprio is combined with info from the vestibular system.. and the visual system (channel-specific)”. The whole hypotheses section also uses the terms “vision” and “proprioception” in a “channel” way. Please just be consistent. And I think introducing some terminology about ‘sensory channels’ or ‘sense organs’ might help.
- Have you done a power analysis? Relatedly – did you have enough trials per condition to find effects in the inevitably noisy children’s data?
- By the end of the section ‘experimental task’ I have understood that you moved them round and had them re-find that position – but I have not understood the various sensory conditions eg IVR on or off, and the point of the markings on the room walls. Put the design/ conditions bit earlier, and clearly state the design. The vision condition is poorly described. Why is it called vision is it seems to be all about removing access to vision? I think you mean that features weren’t visible but optic flow was available through bright, nonspecific markings??

MINOR POINTS

- Vestibular info is key to the study but barely mentioned in the intro.
- Can you spell out more clearly how you arrive at your predictions from previous results (eg according to ref 30, IVR is more disruptive for kids, not adults?).
- I haven’t come across WAIC weights before – can you explain a little more?
- The analyses seem sound but I am not in a position to make really detailed judgment on them.
- You should probably cite literature on balance development e.g. Woollacott, Assainte & more recent ones.
